# Marine mammals and sea turtles listed under the U.S. Endangered Species Act are recovering

**DOI:** 10.1371/journal.pone.0210164

**Published:** 2019-01-16

**Authors:** Abel Valdivia, Shaye Wolf, Kieran Suckling

**Affiliations:** 1 Center for Biological Diversity, Oakland, California, United States of America; 2 Center for Biological Diversity, Tucson, Arizona, United States of America; Deakin University, AUSTRALIA

## Abstract

The U.S. Endangered Species Act (ESA) is a powerful environmental law protecting imperiled plants and animals, and a growing number of marine species have been protected under this law as extinction risk in the oceans has increased. Marine mammals and sea turtles comprise 38% of the 163 ESA-listed marine “species”, which includes subspecies and distinct population segments, yet analyses of recovery trends after listing are lacking. Here we gathered the best available annual abundance estimates for geographically delimited populations of all 62 marine mammal and sea turtle species listed under the ESA. Of these, we chose representative populations of species that were listed before 2012, occur and reproduce in U.S. waters, and have data of sufficient quality and timespan for trend analyses. Thus, we quantitatively analyzed population trends, magnitude of population change, and recovery status for 23 and 8 representative populations of 14 marine mammal and 5 sea turtle species, respectively. Using generalized linear and non-linear models, we found that 18 marine mammal (78%) and 6 sea turtle (75%) populations significantly increased after listing; 3 marine mammal (13%) and 2 sea turtle (25%) populations showed non-significant changes; while 2 marine mammal (9%), but no sea turtle populations declined after ESA protection. Overall, the 24 populations that increased in abundance were from species listed for 20 years or more (e.g., large whales, manatees, and sea turtles). Conservation measures triggered by ESA listing such as ending exploitation, tailored species management, and fishery regulations, and other national and international measures, appear to have been largely successful in promoting species recovery, leading to the delisting of some species and to increases in most populations. These findings underscore the capacity of marine mammal and sea turtle species to recover from substantial geographical population declines when conservation actions are implemented in a timely and effective manner.

## Introduction

Extinction risk for many marine species is increasing as the world’s ocean ecosystems are degraded by pervasive and increasing anthropogenic stressors [1,2] including over-fishing [3], habitat loss and degradation [4], pollution [5], and climate change [6,7]. Recent assessments have identified elevated levels of extinction risk in specific marine taxonomic groups: 14% of seagrasses [8], 16% of mangroves [9], 33% of reef-building corals [10], at least 25% of sharks and rays [11], and 11% of billfish and scombrids (e.g., tunas, bonitos, mackerels) [12]. Although considerably fewer extinctions of marine than terrestrial species have been recorded [1], marine species have a comparably high extinction risk as terrestrial species [13].

The Endangered Species Act (ESA) of the United States is a powerful environmental law, expressly designed to prevent extinction and promote recovery of imperiled species [14]. Under the ESA, a species can be listed as “endangered” if it is in danger of extinction throughout all or a significant portion of its range, and “threatened” if it is likely to become endangered in the foreseeable future (16 U.S.C § 1532(6); (20)). The strength of the ESA lies in its requirement to base decisions on the best available scientific information and its enforceable tools to reduce threats, protect habitat, and restore the abundance and geographic representation of listed species [15]. The ESA’s tools include critical habitat designation, recovery planning with concrete and measurable goals, a science-based consultation process for federal agencies to prevent jeopardizing listed species or adversely modifying their critical habitat, and a prohibition on killing or harming listed species (16 U.S.C. § 1531 et seq.). Species protected under the ESA generally receive tailored federal and state conservation efforts with increased funding for management [16] and thus may have better chances for recovery.

Evaluations of the ESA’s efficacy in preventing extinction and fostering recovery have become more imperative as extinction risks increase [1], available resources for conservation are often limited and mostly insufficient [17], and attacks on the ESA’s effectiveness by political opponents are escalating, with baseless critiques of the law [18]. Analyses to date of the ESA’s performance have consistently concluded that the ESA is highly effective in preventing species extinction [19]. After more than 45 years since the law was enacted in 1973, the ESA has shielded more than 99.5% of the species under its care from extinction [20]. Without the ESA’s protection, an estimated 227 species would have disappeared by 2006 [21].

The ultimate goal of the ESA is to promote the recovery of imperiled species. Numerous analyses have found that species status improves with time since listing, i.e., the longer a species is listed the more its population abundance will increase [22–24]. Populations of species listed as threatened tend to respond faster to protection than populations of endangered species because they generally have higher numbers at the time of listing, requiring relatively shorter time to recover [23,25]. Not surprisingly, species recovery is also associated with effective implementation of the ESA’s tools, including funding for recovery actions [16,22,24,26,27]; presence of a dedicated recovery plan [23,28,29]; progress toward completing recovery goals [30] and designation of critical habitat [30,23,22,24].

Although there were 163 marine “species” listed as threatened and endangered under the ESA (as of August 2018), which includes species, subspecies, and distinct population segments (DPSs) for vertebrates [31], evaluations of the ESA’s track record in protecting marine species are lacking. This is especially evident for the 62 marine mammal and sea turtle ESA species that comprise 38% of currently listed marine taxa [31]. Most studies of population recovery under the ESA are broad analyses of thousands of species [23,32–34] or are tailored to specific terrestrial-related taxa, such as plants [29], anadromous fish [35,36], amphibians [37], or birds [16,25,38,39]. Recent assessments of the status of marine mammal stocks in U.S. waters and global analyses of sea turtle regional management units discuss current population status, but do not analyze recovery trends since ESA listing [40,41]. ESA status reviews by the National Marine Fisheries Service (NMFS) and the U.S. Fish and Wildlife Service (USFWS) are often the only assessments of population trajectories for each listed species [42–45].

The objective of our study was to assess how listed marine mammal and sea turtle species are faring under ESA protections by analyzing populations occurring within U.S. jurisdiction where conservation actions promoted by the law are more robust. Thus, we gathered the best available annual population abundance estimates for all marine mammal and sea turtle species listed under the ESA. Of these, we selected populations of species listed by NMFS and USFWS before 2012, that reproduce or occur in U.S. waters, and had enough quality data to assess population trends during ESA protection (see Table 1 and S1 Table). Thus, we analyzed recovery progress of 23 and 8 representative and geographically delimited populations of 14 marine mammal and 5 sea turtle species. We hypothesize that the assessed populations of marine mammal and sea turtle species listed for more than two decades would be more likely to be recovering than recently listed species. To assess how ESA listing may have influenced population recovery, we calculated population trends (significantly increased, no significant change, or significantly decreased) and magnitude of population change since ESA protection. We discuss conservation actions promoted by ESA listing that may contribute to population recovery, and illustrate this through case studies of three populations of three species: the humpback whale in Hawaii and Alaska, Western Steller sea lion, and the North Atlantic green sea turtle. Our study provides critical information on the recovery potential of imperiled marine mammal and sea turtle populations and supports recent work that highlights a general trend of population increases upon conservation efforts.

**Table 1:** Status of marine mammal and sea turtle populations protected under the ESA included in the analysis.

Common Name	Scientific Name	DPS/Stock/Population	Water	Listed year	ESA status	Years listed	Status change		
**Mammal: Cetacea**									
Beluga whale	*Delphinapterus leucas*	Cook Inlet, Alaska DPS	US	2008	E	9	–		
Blue whale	*Balaenoptera musculus*	Eastern North Pacific Stock	US/F	1970	E	47	–		
Bowhead whale	*Balaena mysticetus*	Western Artic Stock	US/F	1970	E	47	–		
Fin whale	*Balaenoptera physalus*	California-Oregon-Washington Stock	US/F	1970	E	47	–		
		Western North Atlantic Stock	US/F	1970	E	47	–		
Gray whale	*Eschrichtius robustus*	Eastern North Pacific Stock	US/F	1970	E→D	24	1994 –re		
		Western North Pacific DPS	US/F	1970	E	47	–		
Humpback whale	*Megaptera novaeangliae*	Central America DPS	US/F	1970	E	47	–		
		Hawaii DPS	US	1970	E→D	46	2016 –re		
		Mexico DPS	US/F	1970	E→T	47	2016 –dl		
		West Indies DPS	US/F	1970	E→D	46	2016 –re		
Killer whale	*Orcinus orca*	Southern Resident DPS	US	2005	E	12	–		
N. Atlantic right whale	*Eubalaena glacialis*	North Atlantic Population	US/F	1970	E	47	–		
Sei whale	*Balaenoptera borealis*	Eastern North Pacific Stock	US/F	1970	E	47	–		
		Nova Scotia Stock	US/F	1970	E	47			
**Mammal: Carnivora**									
Guadalupe fur seal	*Arctocephalus townsendi*	Guadalupe Island Population	US/F	1985	T	32	–		
Hawaiian monk seal	*Neomonachus schauinslandi*	NW Hawaiian Islands Index Population	US	1976	E	41	–		
Northern sea otter	*Enhydra lutris kenyoni*	Southwest Alaska DPS	US	2005	T	12	–		
Southern sea otter	*Enhydra lutris nereis*	California Population (subspecies)	US	1977	T	40	–		
Steller sea lion	*Eumetopias jubatus*	Western DPS	US/F	1990	T→E	27		1997 –ul
		Eastern DPS	US/F	1990	E→D	23		2013 –re
**Mammal: Sirenia**								
Florida manatee	*Trichechus manatus latirostris*	Florida Population (subspecies)	US	1967	E→T	50		2017 –dl
Antillean manatee	*Trichechus manatus manatus*	Puerto Rico Population (subspecies)	US/F	1970	E→T	47		2017 –dl
**Reptile: Sea Turtles**								
Green turtle	*Chelonia mydas*	Central North Pacific DPS	US/F	1978	T	39		–
		Central West Pacific DPS	US/F	1978	T→E	39		2016 –ul
		North Atlantic DPS	US/F	1978	E→T	39		2016 –dl
		South Atlantic DPS	US/F	1978	T	39		–
Hawksbill turtle	*Eretmochelys imbricata*	U.S. Caribbean Population	US/F	1970	E	47		–
Kemp’s ridley turtle	*Lepidochelys kempii*	Northwest Atlantic, Texas Population	US/F	1970	E	47		–
Leatherback turtle ^a^	*Dermochelys coriacea*	Northwest Atlantic Population	US/F	1970	E	47		–
Loggerhead turtle	*Caretta caretta*	Northwest Atlantic Ocean DPS	US/F	1978	T	39		–

These species were listed before 2012, are found exclusively within United States (US) or within both US and foreign (US/F) waters, have adequate population data that cover at least 40% of the listing period, and the population represents over 50% of the ESA-listed species. Distinct population segment (DPS); listing year; ESA status as endangered (E), threatened (T), delisted (D), or status change (e.g., T→E); and number of years listed are shown. Year of ESA status change due to down-listing (dl) and up-listing (ul); and reason for delisting such as recovered (re) are presented. Several species were listed before 1973 under the Endangered Species Preservation Act of 1966 and the Endangered Species Conservation Act of 1969, which were later replaced by the more comprehensive Endangered Species Act of 1973. See S1 Table for ESA-listed marine mammal and sea turtle species excluded from the analyses. Data as of July 2017 [31].

^a^ The leatherback sea turtle is managed independently in the Atlantic and Pacific Oceans by NMFS. Only Atlantic leatherback sea turtles nest on U.S. beaches, Pacific leatherback were excluded from the analysis.

## Materials and methods

### ESA listed marine mammal and sea turtle species selection

We reviewed the NMFS and USFWS’s endangered and threatened species database *(Environmental Conservation Online System)* and selected all 62 marine mammal and sea turtle “species” currently listed or delisted under the ESA (Table 1, and S1 Table). Under the ESA, the term “species” includes subspecies and distinct population segment (DPSs) (16 U.S.C. § 1532(16). A DPS is defined as a vertebrate fish or wildlife population or a group of populations that is discrete from other populations of the species and is considered significant in relation to the entire species [46]. For example, the humpback whale (*Megaptera novaeangliae*) is currently divided into 14 DPSs under the ESA, of which four DPSs are listed as endangered, one DPS as threatened, and nine DPSs were recently delisted and are considered not at risk [47]. For the designated DPSs of ESA-listed marine mammal and sea turtle species, see Table 1 and S1 Table.

To assess the potential influence of ESA conservation measures on population recovery, we selected populations of extant marine mammal and sea turtle species listed or delisted that meet five criteria: (1) from species listed before 2012 to provide a minimum timeframe of post-listing population data for conservation measures to be applied; (2) occurrence and reproduction in U.S. waters, i.e., excluding species or populations that occur and reproduce (e.g., nesting for sea turtles) in foreign waters/grounds where the ESA provides fewer protections [48]; (3) with enough reliable abundance data to determine population-level trends, i.e., at least three data points within 10 years, which is generally recommended for determining population change in ESA endangered and threatened species [49] and has been used for marine mammals [50] and sea turtles [51]; (4) with population data covering at least 40% of the ESA listing period, which we considered adequate for determining population trends after ESA listing; and (5) with populations that numerically represents over 50% of the abundance of the listed species, subspecies, DPS, or marine mammal stock and sea turtle regional management unit (RMU) within U.S. jurisdiction. For example, most green sea turtles of the North Atlantic DPS within U.S. jurisdiction nest in Florida and thus nest counts in Florida were used to represent this DPS. To delimit a population in our study after data selection, we used abundance data consistently collected over time in U.S. waters (including nesting/foraging grounds) in geographically delimited areas such as DPSs under the ESA, stocks under the Marine Mammal Protection Act (MMPA), and RMUs for sea turtles [52] (S1–S3 Figs). As a result, population trend calculations are likely representative of the status of the listed species, subspecies, DPS, stock or RMU within U.S. jurisdiction even though these may be comprised of several populations. We identified 31 representative populations that met our selection criteria, totaling 23 and 8 populations of 14 marine mammal and 5 sea turtle species, respectively (Table 1 and S1 Table). Of the 43 marine mammal and sea turtle ESA-listed species (including subspecies and DPSs) that did not meet our selection criteria, 72% do not occur or reproduce in U.S. waters. For approximate geographic distribution of each population, see S1–S3 Figs in supporting information.

We also evaluated changes in species protection status. Species can be listed under the ESA as endangered or threatened. The ESA defines an endangered species as “in danger of extinction throughout all or significant portion of its range” while threatened species are “likely to become endangered in the foreseeable future throughout all or significant portion of its range” (16 U.S.C. § 1532(6) and (20)). For several species, the protection status (i.e., endangered or threatened) changed since the species was first listed at the global population level, and a few species were divided into DPSs (Table 1 and S1 Table). For the purpose of our study, we used the most current ESA protection status but the original year that the species was protected (Table 1).

### Data compilation and availability

We collected information and population-level abundance estimates for ESA-listed marine mammal and sea turtle species from published papers and government reports. Main data sources included NMFS and USFWS technical memorandum and administrative reports, U.S. marine mammal stock assessment reports, species recovery plans, five-year status reviews, and primary sources from peer-reviewed scientific journals (S1 Dataset). When possible, we collected abundance data up to 2017 or to the most recently available population-level estimate. For populations that occur and reproduce in both U.S. and foreign waters, we used datasets from surveys that occurred in waters and nesting/foraging grounds under U.S. jurisdiction.

Population abundance estimates came from a variety of survey methodologies (aerial, land, and ship-based surveys), mark-recapture population modeling, extrapolated data based on sex ratios, and photo-identification models (S1 Dataset). For marine mammals, population abundance comprised the total number of individuals including adults, juveniles, and pups or calves. For sea turtles, we used number of nests on nesting beaches, number of nesting females, or number of individuals in foraging grounds to determine population trends. The number of nesting females and number of nests are common metrics for monitoring and evaluating population status of sea turtles [51].

Estimate bias and errors in population abundance obtained from data sources were variable among populations and even within the same population over time. For example, survey effort and methodologies changed over time and population estimates have been calculated using different approaches over the years for the same population (e.g., traditional population abundance models, Bayesian population models, or habitat-based density models). Thus, when available, each data point was accompanied with information on data collection methodology, error information (e.g., coefficient of variation), and data estimation reliability (S1 Dataset). Time-series of population abundance for each species were carefully constructed to ensure all annual data points were derived from adequate and quantitative methodologies with comparable survey efforts.

### Population trends and magnitude of change

For each marine mammal and sea turtle representative population, we calculated the population trend (as percentage change per year) and the magnitude of population change (as percentage change) after ESA listing based on the predicted distributions from the best and final fitted generalized linear or non-linear models (Table 2 and S2 Table). Population trajectories were classified as significantly increasing, non-significant change (non-significant slope), or significantly decreasing as in Magera et al. [50]. Recovering populations were defined as those that significantly increased in abundance after ESA listing, independently of whether or not they were on track to meet the recovery criteria for downlisting or delisting found in recovery plans. Populations with non-significant trends were not classified as “stable” as in other studies [40]. This was because determining population stability over time requires further assessment of the accuracy of annual population estimates (e.g., the confidence intervals), which were often not available. Analysis of the magnitude of population change from estimated historical baselines was also not performed because this has been described elsewhere [50,53,54].

**Table 2:** Linear model and ANOVA results of the relationship between time since ESA listing and population trends (increasing, non-significant, decreasing) for marine mammal and sea turtle populations.

**Linear model**	**Estimate**	**Std. Error**	**t value**	**Pr(>|t|)**
(Intercept)	24.500	6.652	3.683	0.00098 ***
Non-significant trend	0.100	7.871	0.013	0.98995
Increasing trend	15.250	6.924	2.202	0.03603 *
**ANOVA**	**DF**	**Mean Sq**	**F value**	**Pr(>F)**
Trend	2	624.29	7.0535	0.00331 **
Residuals	28	88.51		

The decreasing trend was used as reference for the linear model.

Significant codes are ‘***’ for p<0.001, ‘**’ for p< 0.01 and ‘*’ for p< 0.05.

### Data analysis: Population trajectories and model selection

To assess population trajectories after listing we used several types of models including linear models (*lm*), generalized linear models (*glm*), generalized least square models (*gls*), or generalized additive models (*gam*) in which population abundance estimates were modeled by running time in years (S2 Table). Because population trends were species specific, we used several family distributions and error links for each of the population-level models (S2 Table). For each population, we performed a comprehensive exploratory analysis using all model types and possible combinations of families and links with and without a log transformation of the abundance estimates. In several *gls* models we added correlation and variance structures to account for potential temporal autocorrelation among years and variation in the data (S2 Table). Improvement in model fit was evaluated through theoretical model inference based on Akaike’s Information Criterion (AIC) [55], and comparing adjusted regression (r-squared) parameters when available [56]. Final model selection was based on a multi-model inference approach using AICc corrected for small samples [57]. See supporting information for final model details (S2 Table). All calculations and graphing were performed in R version 3.3 [58] using the packages *nlme v*.*3*.*1–131* for generalized least squared models [59]; *gam v*.*1*.*14–4* for generalized additive models [60]; *MuMIn v*.*1*.*15*.*6* for multi-model inference [61]; and *ggplot2 v*.*2*.*2*.*1* for data visualizations [62]. The dataset with specific data sources and references (S1 Dataset), and the R code of the analysis (S1 R Code) are provided in supporting information.

## Results

### Status of ESA-listed marine mammal and sea turtle species

Protection status for 10 out of the 31 representative populations analyzed in our study changed since they were first listed, with eight of the 10 improving in status. Four ESA-listed species were downlisted by NMFS and USFWS from endangered to threatened: the Mexico DPS of humpback whale in 2016; the Florida manatee (*Trichechus manatus latirostris*) and the Antillean manatee (*Trichechus manatus manatus*) subspecies in 2017; and the North Atlantic DPS of green sea turtle in 2016 (Table 1). Four ESA-listed species were delisted because NMFS determined they have recovered: the Eastern North Pacific stock of gray whale (*Eschrichtius robustus*) in 1994, two DPSs of humpback whales (Hawaii and West Indies) in 2016, and the Eastern Pacific DPS of Steller sea lion in 2013 (Table 1). Two ESA-listed species were uplisted from threatened to endangered: the Western Pacific DPS of Steller sea lion (*Eumetopias jubatus*) in 1997, and the Central West Pacific DPS of green sea turtle in 2016 (Table 1).

### Population trends and magnitude of change

**Fig 1:**
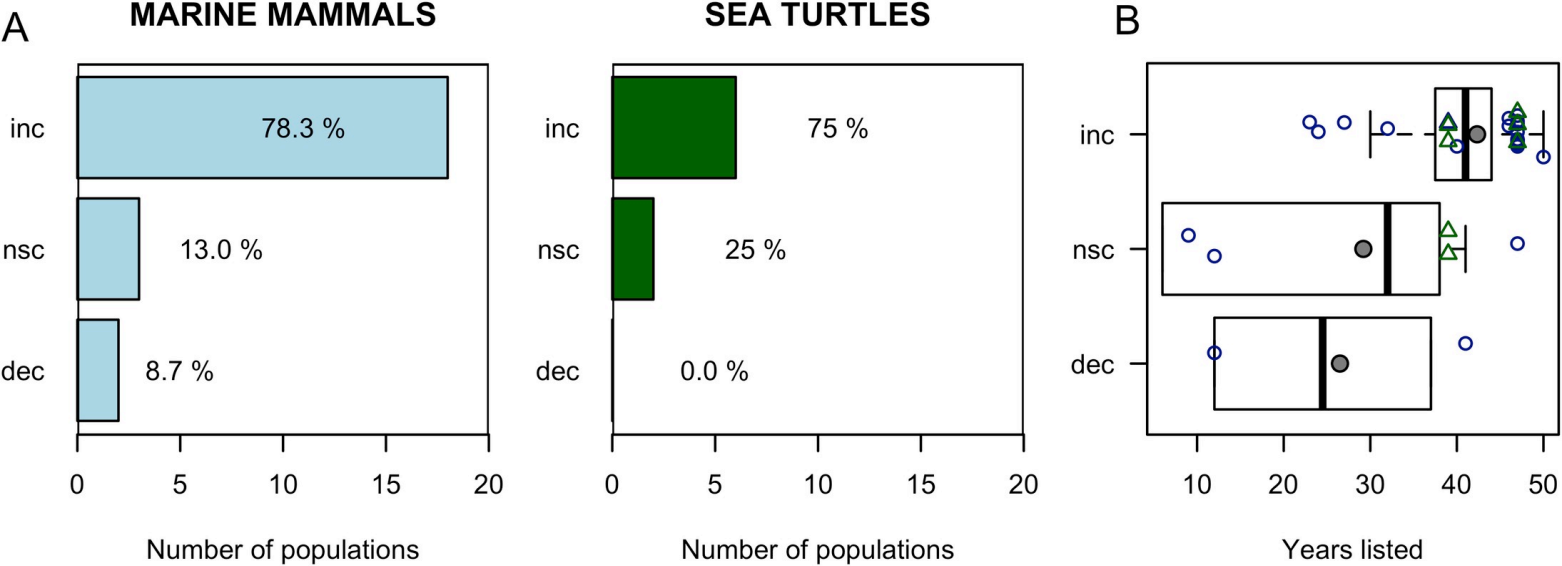
Number and percentage of marine mammal and sea turtle populations protected under the ESA that significantly increased (inc), non-significantly change (nsc), and significantly decreased (dec) after listing. **(A)** Calculations were based on 23 marine mammal and 8 sea turtle representative populations of ESA-listed species that met our selection criteria. **(B)** Relationship between population trend and time since listing for marine mammal (blue circles) and sea turtle (green circles) populations. Black line is the median and grey circle the mean.

Overall, 18 out of 23 of marine mammal populations (~78%) and 6 out of 8 sea turtle populations (75%) analyzed that met our selection criteria significantly increased in abundance after ESA listing (Fig 1A). Representative populations of three marine mammals (~13%) and two sea turtles (~25%) showed non-significant change. Only two marine mammal populations (~9%), but no sea turtles significantly declined after ESA protection (Fig 1A). Marine mammal and sea turtle populations that significantly increased were from species listed between two to five decades and increasing population trends was positively associated with time since listing (p = 0.036). In contrast, there was no association with listing time for populations that showed non-significant trend or that declined in abundance (Fig 1B; Table 2). There was also no association between the time series length (i.e., number of data points) and the number of populations that increased, did not change, or decline (F-value = 1.525, p = 0.235). Out of the 24 populations that significantly increased, 50% were from species listed as endangered, 33% as threatened, and 17% were delisted, indicating that population increases occurred independent of whether a species was classified as threatened or endangered (Tables 1 and 2).

**Fig 2:**
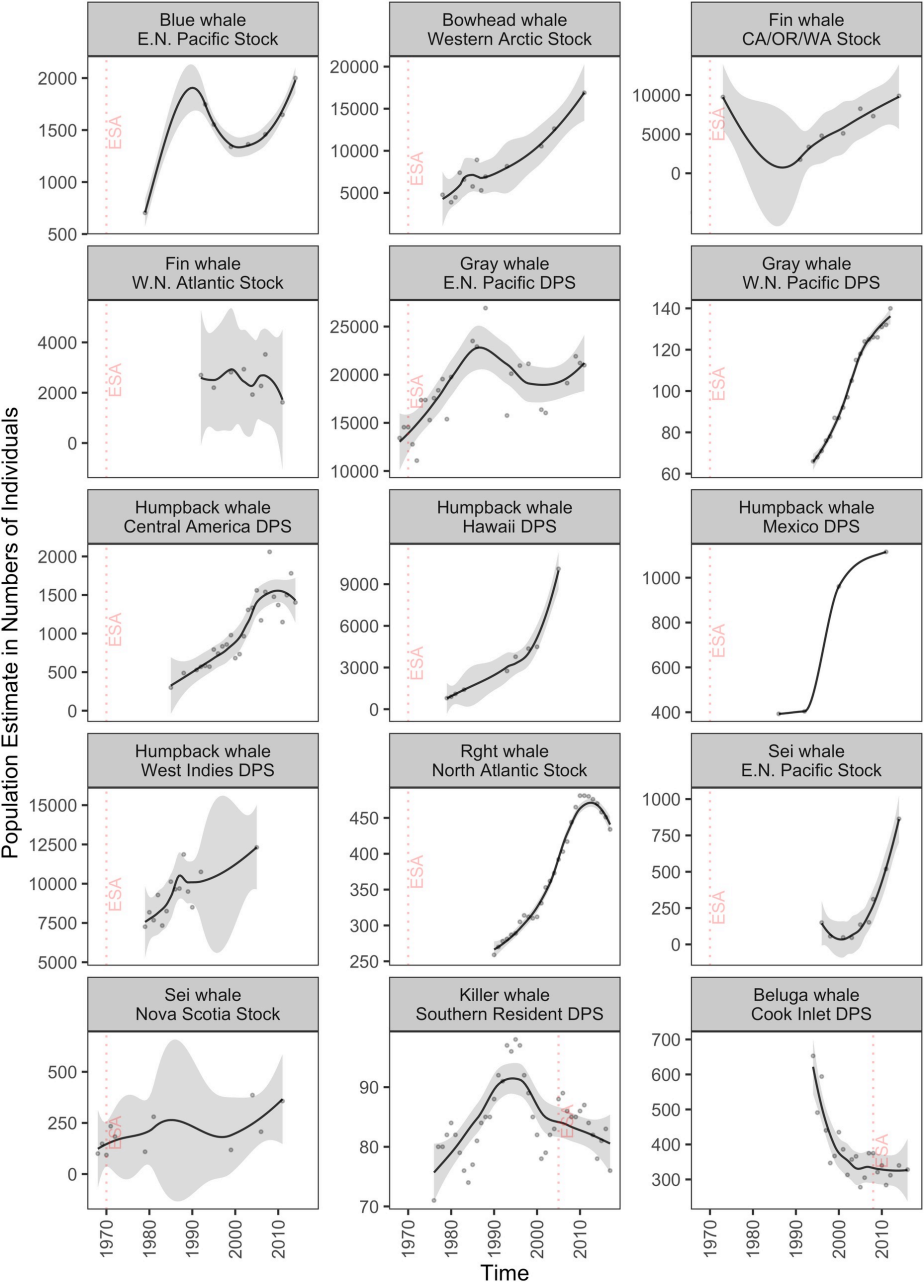
Population-level trends of cetacean marine mammals listed under the ESA. Trend lines (gray area: 95% confidence interval) are loess curves with span of 0.5 to aid in visual representation. Grey dots are estimated number of individuals. Panels are organized by decreasing length of time listed and then in alphabetical order based on species names. Dashed vertical red lines indicate the year of ESA listing. For population selection criteria see methods; for protection status see Table 1; and for results of fitting models see S2 Table. Abbreviations are CA/OR/WA: California/Oregon/Washington; E.N.: Eastern North; and W.N.: Western North.

**Fig 3:**
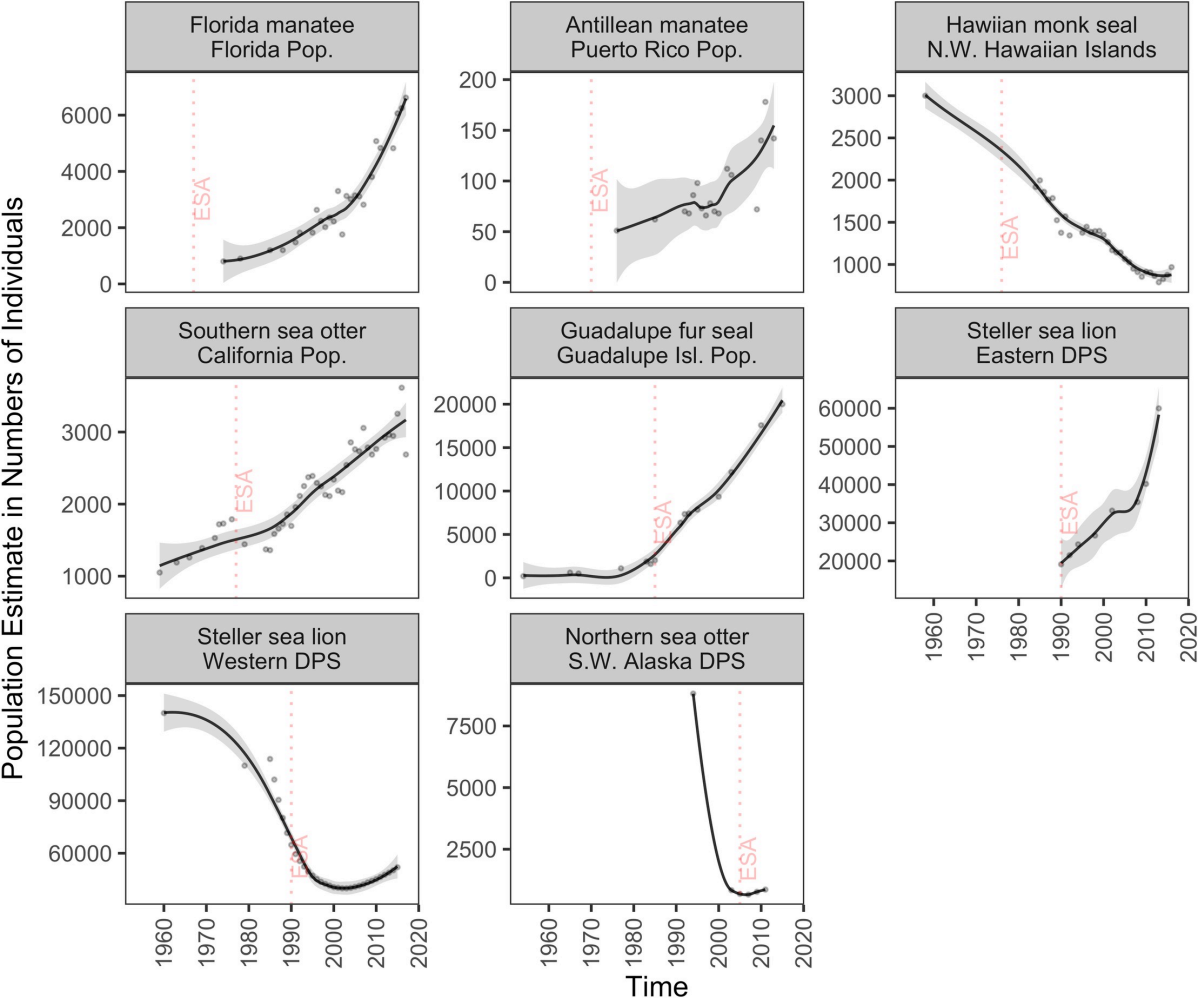
Population-level trends of non-cetacean marine mammals listed under the ESA. Trend lines (gray area: 95% confidence interval) are loess curves with span of 0.5 to aid in visual representation. Grey dots are estimated number of individuals. Panels are organized by decreasing length of time listed. Dashed vertical red lines indicate the year of ESA listing. For population selection criteria see methods; for protection status see Table 1; and for results of fitting models see S2 Table. Abbreviations are DPS: Distinct Population Segment; Pop.: Population; N.W. North Western; and S.W: Southwest.

Most marine mammal populations that significantly increased after ESA listing had substantial population growth (Figs 2 and 3; Table 3). Several populations of large whale species increased in numbers from ~3% to ~43% per year, often doubling to quadrupling their initial population estimates (Table 3). For example, all four DPSs of humpback whales analyzed in our study showed substantial population increases (Fig 2; Table 3). In fact, the Hawaiian DPS of humpback whale reached over 10,100 individuals in 2005 from only 800 individuals estimated in 1979 (Fig 2; Table 3). NMFS subsequently delisted it from the ESA in 2016 (Table 1). While most large whale populations trended toward recovery, the critically endangered population of the North Atlantic right whale (*Eubalaena glacialis*) increased at 4.2% per year from 270 to 481 whales between 1990 and 2010, but declined to an estimated 434 whales between 2010 to 2017 due to entanglement in fishing gear and vessel collisions (Fig 2; Table 3 and S2 Table).

**Table 3:** Trends and magnitude of change of selected marine mammal and sea turtle populations protected under the ESA. Population (Pop.) trends (significantly increased ↑, non–significant change →, significantly decreased ↓) are based on population-specific models and time periods are shown. Current population trends (% per year) and magnitude of population change (%) were calculated based on available data after listing. First and last population abundance estimates for the time period are shown for reference. DPS: Distinct Population Segment; NWR: National Wildlife Refuge.

ESA Species (DPS/Stock/Location)		Time period (years)	Pop. trend (sign)	Pop. trend (% yr^-1^)	Pop. change (%)	First pop. estimate (No.)	Last pop. estimate (No.)
**Cetacean**							
Beluga whale (Cook Inlet DPS)		08–14	→	– 0.44	– 8.8	375	340
Blue whale (Eastern North Pacific Stock)		79–14	↑	+ 4.99	+ 174.5	705	2,000
Bowhead whale (Western Arctic Stock)		78–11	↑	+ 8.34	+ 273.1	4,765	16,892
Fin whale (California-Oregon-Washington Stock)		91–14	↑	+ 13.34	+ 306.9	1,744	9,892
Fin whale (Western North Atlantic Stock)		92–11	→	– 0.75	–14.2	2,700	1,618
Gray whale (Eastern North Pacific Stock)		70–11	↑	+ 1.28	+ 52.6	14,553	20,990
Gray whale (Western North Pacific Stock)		94–12	↑	+ 6.22	+ 111.9	66	140
Humpback whale (Central America DPS, California + Oregon)		85–14	↑	+ 15.18	+ 440.2	300	1,403
Humpback whale (Hawaii DPS, Hawaii winter)		79–05	↑	+ 42.86	+ 1,114.3	800	10,103
Humpback whale (Mexico DPS, Southeast Alaska to Alaska Peninsula)		86–11	↑	+ 13.40	+ 334.4	393	1,115
Humpback whale (West Indies DPS)		79–05	↑	+ 3.00	+ 78.0	7,260	12,312
Killer whale (Southern Resident DPS)		05–17	↓	– 0.93	– 11.2	88	76
North Atlantic right whale (North Atlantic)		90–10	↑	+ 4.20	+ 84.0	270	481
		10–17	↓	– 1.37	– 9.6	481	434
Sei whale (Eastern North Pacific Stock)		96–14	↑	+ 33.09	+ 595.6	150	864
Sei whale (Nova Scotia Stock)		70–11	↑	+ 1.98	+ 81.4	93	357
**Carnivora**							
Guadalupe fur seal (Guadalupe Island, Mexico)		85–15	↑	+ 14.84	+ 905.4	2,017	20,000
Hawaiian monk seal (NW Hawaiian Islands)		85–13	↓	– 2.04	– 57.0	1,997	789
		13–16	↑	+ 5.72	+ 22.9	789	968
Northern sea otter (Southwest Alaska DPS, Attu, Amchitka, Adak, Kiska Islands)		05–11	→	+ 5.06	+ 30.3	687	863
Southern sea otter (California)		79–17	↑	+ 3.02	+ 114.7	1,443	2,688
Steller sea lion (Eastern DPS, California to Southeast Alaska)		90–13	↑	+ 5.79	+ 133.2	19,103	59,968
Steller sea lion (Western DPS, Alaska)		90–03	↓	– 3.04	– 39.4	64,761	39,963
		03–15	↑	+ 2.34	+ 28.1	39,963	52,009
**Sirenia**							
Florida manatee (Florida)		74–17	↑	+ 17.14	+ 737.3	800	6,620
Antillean manatee (Puerto Rico)		76–13	↑	+ 4.75	+ 175.8	51	142
**Sea Turtles**							
Green turtle (Central North Pacific DPS, East Island, French Frigate Shoals, HI)^1^		78–16	↑	+ 12.66	+ 480.9	101	88
Green turtle (Central West Pacific DPS, Guam waters)^2^		78–10	→	+ 7.46	+ 238.6	92	299
Green turtle (North Atlantic DPS, Florida index beaches)^3^		89–16	↑	+ 75.71	+ 2,044.2	464	2,978
Green turtle (South Atlantic DPS, Buck Reef NWR + Sandy Point NWR + Jack, Isaac, and East End Bays, VI)^3^		82–15	↑	+ 104.2	+ 3,439.1	31	931
Hawksbill turtle (U.S. Caribbean population, Mona Island, Puerto Rico)^3^		74–15	↑	+ 22.64	+ 928.5	177	1,328
Kemp’s ridley turtle (Texas)^3^		79–17	↑	+ 284.2	+ 11,083.8	1	353
Leatherback turtle (Atlantic DPS, Florida + Puerto Rico + Sandy Point NWR, VI)^3^		84–16	↑	+ 32.25	+ 1,032.2	368	3,625
Loggerhead turtle (NW Atlantic DPS, Peninsular Florida index beaches)^3^		89–16	→	+ 1.16	+ 31.4	39,083	65,807

^1^ Number of nesting females

^2^ Number of individuals

^3^ Number of nests.

Populations of non-cetacean marine mammal species also significantly increased in abundance at relatively high growth rates since ESA protection. Notably, the population of the Guadalupe fur seal (*Arctocephalus townsendi*) increased about nine times at ~15% per year since the species was listed in 1985 (Fig 3; Table 3). The California population of the Southern sea otter (*Enhydra lutris nereis*) approximately doubled in numbers and it is likely to reach the demographic recovery criteria in the coming years (Fig 3; Table 3). The Eastern DPS of Steller sea lion (*Eumetopias jubatus*) tripled its population at ~6% per year since 1990, reaching its recovery criteria of ~60,000 individuals in 2013, and was subsequently delisted from the ESA (Fig 3; Table 3). Also, both populations of the Florida and Antillean manatee subspecies increased approximately eight and three times (~17% and ~5% per year), respectively, in the past 40 years (Fig 3; Table 3); and USFWS downlisted them from endangered to threatened in 2017 (Table 1).

Representative populations of five marine mammal species analyzed in our study did not increase in abundance. Three representative populations of three marine mammal species showed non-significant population change after listing: Western North Atlantic stock of the fin whale (*Balaenoptera physalus*), Cook Inlet beluga whale (*Delphinapterus leucas*) DPS (Fig 2; Table 3 and S2 Table), and Southwest Alaska DPS of the northern sea otter (*Enhydra lutris kenyoni*) (Fig 3; Table 3 and S2 Table). In contrast, two marine mammal populations significantly declined after ESA listing: the critically endangered Southern Resident killer whale (*Orcinus orca*) and the Hawaiian monk seal (*Neomonachus schauinslandi*). Southern Resident killer whales declined at– 0.93% per year since listing in 2005, when the population had 88 individuals (Fig 2, Table 3). This population suffered major declines after a record high of 98 individuals in 1995, and the last population survey estimated 76 individuals as of December 2017, a 30-year low (Fig 2; Table 3). Total abundance of Hawaiian monk seals from six index subpopulations in the Northwestern Hawaiian Islands significantly declined from 1,997 individuals in 1985 to 789 seals in 2013 at approximately– 2% per year (Fig 3; Table 3). However, the population had increased to 968 seals by 2016 (Table 3).

**Fig 4:**
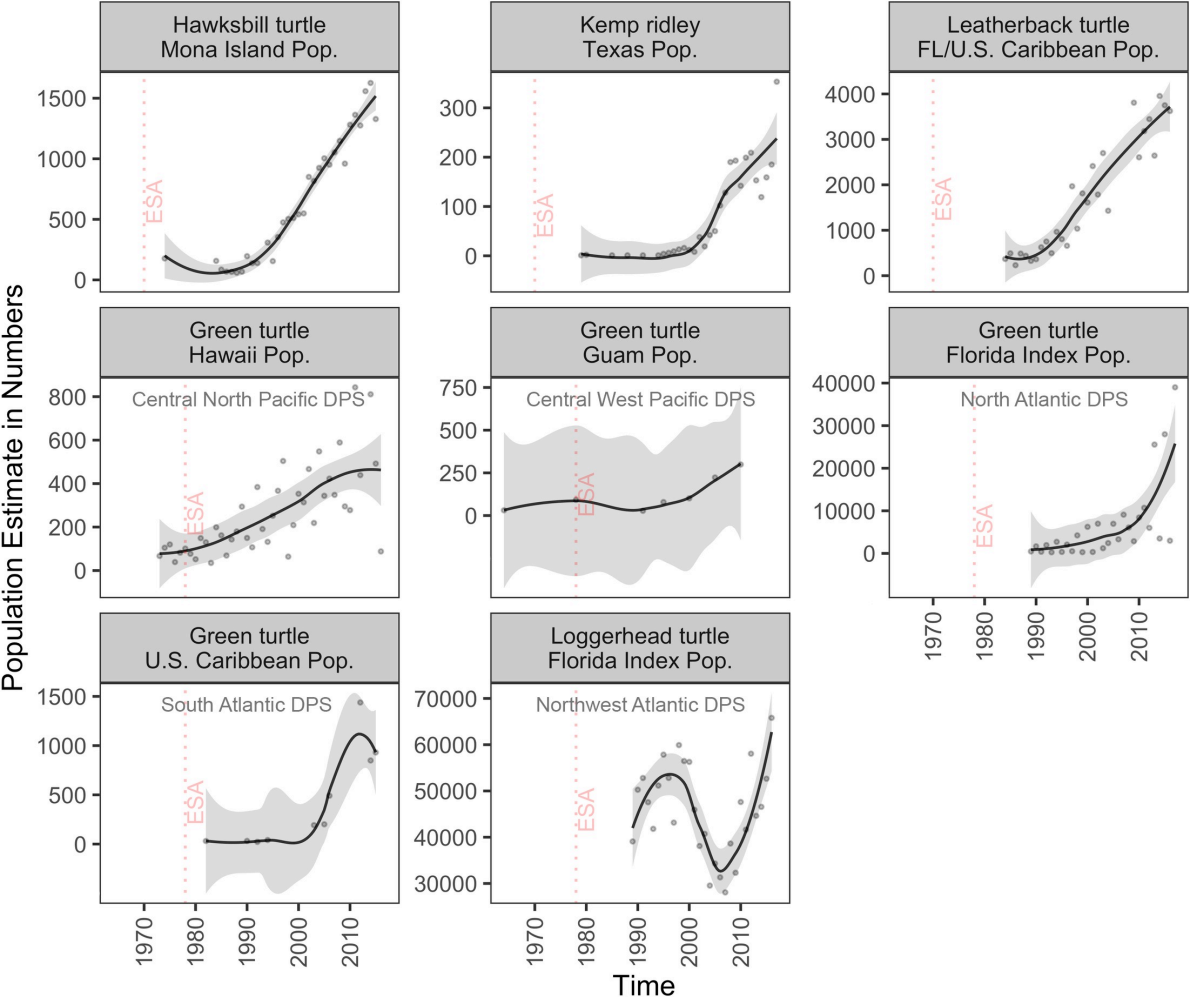
Population-level trajectories of sea turtles listed under the ESA. Trend lines (gray area: 95% confidence interval) are loess curves with span of 0.5 to aid in visual representation. Grey dots are estimated number of nests, except number of nesting females (green turtle, Hawaii population), and number of individuals (green turtle, Guam population). Panels are organized by decreasing length of time listed. Dashed vertical red lines indicate the year of ESA listing. For population selection criteria see methods; for protection status see Table 1; for results of fitting models see S2 Table; and for DPS of each population (Pop.) see Table 3.

Six of the eight populations of the five sea turtle species analyzed in our study significantly increased after ESA listing (Fig 4; Table 3 and S2 Table). Estimates of the number of individuals, nesting females, and number of nests in nesting beaches of representative populations of green, hawksbill, Kemp’s ridley, and Atlantic leatherback sea turtle species showed that these populations increased at considerably high growth rates (~13% to ~284% per year) for several decades, depending on initial estimates (Fig 4; Table 3 and S2 Table). For example, the number of nesting females of green sea turtle at East Island of the French Frigate Shoals in Hawaii (from the Central North Pacific DPS) increased at ~13% per year from 101 individuals in 1978 to 492 nesting females in 2015 (Fig 4; Table 3). The number of nests of the green sea turtle nesting population across Florida statewide beaches (from the North Atlantic DPS) increased at ~76% per year from 62 nests in 1979 to a record high of 37,341 nests in 2015 (Fig 4; Table 3). Due the strong recovery of green sea turtles across Florida, NMFS and USFWS downlisted the entire North Atlantic DPS from endangered to threatened in 2016 (Table 1). Similarly, the number of nests of the hawksbill turtle nesting population at Mona Island in Puerto Rico increased at over 22% per year from 177 in 1974 to a record high of 1,626 nests in 2014 (Fig 4; Table 3). Notably, the Atlantic leatherback nesting populations within U.S. jurisdiction have also experienced a considerable rebound, and the combined number of nests across Florida, Puerto Rico, and the Virgin Islands, significantly increased after ESA listing (Fig 4; Table 3).

Among the sea turtle populations analyzed in this study, models were not able to detect significant linear trends for the Central West Pacific DPS of the green turtle (Guam waters), and the Northwest Atlantic DPS of the loggerhead turtle (*Caretta caretta*) across the Florida peninsula (Fig 4; Table 3 and S2 Table). The best models for the number of nests of loggerhead turtles across index beaches of the Florida peninsula described a non-linear relationship where the number of nests substantially fluctuated since 1989, with a record high of 65,807 in 2016 (Fig 4).

## Discussion

Most representative populations of marine mammal and sea turtle species protected under the ESA that met our selection criteria significantly increased after listing, indicating population recoveries. Significant population increases for most marine mammal and sea turtle species after ESA protection demonstrate the capacity of these taxa to rebound from drastic population declines after decades of exploitation, habitat degradation, and other threats, once effective conservation measures are in place. Our analyses confirm the hypothesis that populations of ESA-listed marine mammal and sea turtle species are more likely to be recovering the longer they stay protected under the law, regardless of whether they are listed as threatened or endangered. Previous studies support these findings for a variety of terrestrial taxa, marine birds, and anadromous fishes [16,19,23,25,39,63]. Thus, our results provide critical information on the recovery time for depleted marine mammal and sea turtle populations that can inform planning for effective management and ground expectations for recovery success.

Our results also support previous studies that highlight the capacity of marine mammals and sea turtles to rebound from decades of exploitation after coordinated national and international conservation efforts [40,41,50,64,65]. For example, a recent analysis found that 12 of 17 (70%) sea turtle regional management units globally have shown an upward significant trend, with even small populations showing signs of recovery [41]. Population recovery of sea turtles have been linked to effective protection of nesting females and eggs, as well as bycatch reduction [41,42]. Similarly, out of 92 spatially non-overlapping marine mammal populations around the world, 42% have shown significant population increases and 28% have shown non-significant change [50].

Here we discuss how the protections of the ESA and complementary conservation measures have been important for the recovery of ESA-listed marine mammal and sea turtle populations occurring in U.S. jurisdiction, and illustrate specific examples through three case studies. The ESA’s prohibitions on commercial exploitation paired with the implementation of widespread conservation measures such as interagency consultation, recovery plans and critical habitat designations have been crucial to mitigating threats that affect marine mammals and sea turtles [34,66]. Between the 18^th^ to early 20^th^ century these groups were substantially depleted [4,54,67,68], in a few cases to extinction such as the Steller’s sea cow [69] and the Caribbean monk seal [70,71]. Marine mammal and sea turtle populations have greatly benefited from a major change from resource exploitation (e.g., whaling, hunting, egg harvesting) to conservation measures that protect them from direct and indirect harm [72].

For the large whales, ESA protections facilitated the recovery of populations that were severely depleted by commercial whaling by reducing key threats such as ship strikes, entanglement in fishing gear, and pollution [44,66,73–76]. For example, ESA protection led to the establishment of vessel speed limits and restrictions on approaching whales too closely to lower the likelihood of death and injury from vessel strikes [77–79]. By triggering a depleted designation under the U.S. Marine Mammal Protection Act (MMPA), ESA marine mammal listings have prompted the implementation of take reduction plans to reduce injury and death from fisheries entanglement that require gear modifications, time and area closures, and vessel observers [40,80]. ESA regulations have also helped to limit acoustic harms to whales and other marine mammals by restricting U.S. military use of sonar and explosions in biologically important habitat areas around Hawaii Islands and Southern California [81]. However, entanglement in fishing gear and collisions with vessels continue to be major threats to vulnerable large whale species [82]. For example, the North Atlantic right whale population has suffered a significant decline in the last decade due to entanglement in fishing gear and vessel collisions, threats that are compromising the recovery of this critically endangered population [82–84].

For sea turtles within U.S. waters, ESA protections have been instrumental in reducing primary threats from harvest, fishery bycatch, habitat destruction, predation on nesting beaches, and trade. The ESA’s prohibitions on harvesting sea turtles and their eggs has virtually eliminated this key threat–historically the principal cause of sea turtle population declines–in U.S. turtle nesting and foraging grounds [42,43]. ESA listing prompted regulations that have reduced sea turtle bycatch mortality in commercial fisheries by requiring gear modifications (e.g., turtle excluder devices in trawl fisheries, circle hooks in longline fisheries, modifications to pound net leaders), time and area closures, bycatch limits, changes to fishing practices, and monitoring programs [85–88]. ESA-prompted reductions in off-road-vehicle use and night lighting on nesting beaches have promoted nesting activity [89,90], as has protection of important turtle nesting beaches such as National Wildlife Refuges (NWR) on the Atlantic coast (e.g., Archie Carr NWR, Florida) and the U.S. Caribbean (e.g., Culebra NWR, Puerto Rico; Sandy Point NWR, U.S. Virgin Islands) [42,43,91]. Among the sea turtle species with remarkable recovery success are the nesting populations of leatherbacks, green turtles and loggerhead turtles, especially across Florida [42,43,92]. In addition, ESA protections have facilitated federal and state agencies (e.g., National Park Service, Florida Fish and Wildlife Conservation Commission) to contribute funding and support conservation efforts including species reintroductions, e.g., Kemp’s ridley turtles in Texas [93], and volunteer monitoring and scientific data collection on most sea turtle nesting beaches across the U.S. (e.g., Florida Statewide and Index Nesting Beach Survey program).

Importantly, the successful conservation of sea turtles has also relied on international cooperation, multinational agreements, and in-country efforts to effectively protect these highly migratory species [41,42]. Several international conservation efforts such as the Convention on International Trade in Endangered Species of Wild Fauna and Flora (CITES) which listed sea turtles in 1981, the 1996 Inter-American Convention (IAC) for the protection and conservation of sea turtles, and the Convention for Migratory Species (CMS) which listed sea turtles in 1983, among others, have also been important in the reduction of threats, especially trade of sea turtle products, harvest, and incidental bycatch [42,43,94,95]. Regional and national legislation, harvesting bans, and local conservation efforts by non-governmental groups have given protection to sea turtle species in neighboring countries that may have contributed to recovery of U.S. nesting populations [42].

Two marine mammal populations that did not significantly change were from species listed relatively recently (< 15 years). The Cook Inlet DPS of beluga whale in Alaska was listed in 2008 and the Southwest Alaska DPS of the northern sea otter was listed in 2005. Conservation measures for these two species were developed relatively recently and ongoing threats have not been mitigated [96,97]. It is likely that these populations will require more time under ESA protection as well as the adoption of robust conservation measures. In contrast, populations of one marine mammal and two sea turtle species listed for several decades showed non-significant change. The lack of significant population changes in the Western North Atlantic stock of fin whale and the Central West Pacific DPS of green turtle may be related to lack of statistical power to detect a trend in abundance as confidence intervals of population estimates were relatively large (Figs 2 and 4; S1 Table) [42,98]. Alternatively, the populations of these species may be stable, but further population estimates are needed to determine stability [42,98]. Finally, fluctuations in the number of nests of the nesting population of loggerhead turtle across Florida beaches have been strongly correlated with ocean conditions associated with long term climate forcing such as the Atlantic Multidecadal Oscillation [99].

Endangered marine mammal species with relatively low population abundance that significantly declined after listing (e.g., Southern Resident killer whale and Hawaiian monk seal) or showed non-significant change (e.g., Cook Inlet beluga whale) require urgent conservation attention. NMFS already recognizes these species among those most at-risk of extinction in the immediate future and they are considered recovery priorities because of rapid population declines [100]. These species face several similar regional anthropogenic threats including prey reduction due to fishing, habitat degradation, toxic pollutants, disturbance from boat traffic and marine noise, fishery interactions, as well as global threats associated with climate change and ocean regime shifts that affect food availability [101–105]. In particular, food limitation has been recognized as a key driver of lower body condition, pregnancy failures, calf/pup and juvenile mortality, and lack of population recovery [105–109]. Numerous conservation measures addressing anthropogenic stressors have been developed for these species and are delineated in recovery plans [97,110,111]. For example, NMFS established regulations to protect killer whales in Washington waters from vessel impacts in 2011 [112]. For Hawaiian monk seals, entanglements in fishing gear, fishery interactions, and other human-caused mortalities (e.g., intentional killing) have been reduced since ESA listing, especially across the inhabited Main Hawaiian Islands [111,113]. In fact, after more than 50 years of continued decline, the range-wide population seems to have steadily increased in numbers since 2013, reaching approximately 1,400 seals in 2016 [114]. Recently, stronger conservation measures have been developed in high-priority action plans that focus efforts and resources to reduce threats and stabilize population declines [100]. The outcomes of these conservation efforts will require time to be realized, although the compounding effects of climate change stressors may compromise the ability of these endangered species to rebound.

### Case studies illustrate the recovery benefits of ESA listing

#### Hawaii DPS of humpback whale

The Hawaii DPS of humpback whale was delisted by NMFS in 2016 based on its strong population growth and the mitigation of key threats (NMFS 2015). Whales in this population use the waters surrounding the main Hawaiian Islands for mating and calving and migrate to feeding in areas off Southeast Alaska and northern British Columbia. The size of the population in Hawaiian waters increased from 800 individuals in 1979 to more than 10,000 individuals in 2005, with the recent population growth rate estimated around 6% (NMFS 2015). ESA listing in 1970 prompted conservation measures in Hawaii and Alaska to reduce key threats to recovery. ESA regulations restricted vessels in Hawaiian and Alaskan waters from approaching whales within 100 yards, prohibited disrupting normal behaviors, and required slower vessel speeds to reduce the likelihood of ship strikes and minimize human disturbance [115,116]. ESA listing also prompted coordinated federal and state efforts to reduce whale entanglements in fishing gear through the Hawaiian Islands Disentanglement Network and Alaska Response Network. The threatened status of humpback whales also provided impetus for the designation of the 1,400 square-mile Hawaiian Islands Humpback Whale National Marine Sanctuary in 1992 to protect humpback whales and their habitat [117].

#### Western DPS of Steller sea lion

Population abundance of the Western DPS of Steller sea lion, which ranges from Eastern Gulf of Alaska to the Western Aleutian Islands and Bering Sea [118], significantly increased over the past 13 years (Fig 3). This species has shown a tremendous population recovery despite years of overexploitation (for their fur, meat, and oil), indiscriminate killing, and decades of habitat degradation, ship strikes, and fishery interactions [119]. Abundance estimates of the Western DPS declined from 140,000 to 110,000 individuals between 1960 and 1979 in rookeries and haul-outs across Southwest Alaska [119]. Total counts continued to decline at 15% per year in the late 1980s, prompting NMFS to list the Western DPS as threatened throughout the entire range in 1990 (NMFS 2008) and to uplist it to endangered in 1997 because of continued declines during the 1990s [120]. Population abundance stabilized in the early 2000s [121,122] with the lowest population estimate in 2003 [118]. Notably, population abundance significantly increased at 2.34% per year from 2003 to 2015 (Table 3).

Conservation efforts under both the ESA and the MMPA such as designation of protective zones, critical habitat designation, fishery regulations for prey species, and local regulations around major rookeries and haul-outs have likely contributed to the population recovery success [119]. NMFS implemented several fishery management measures (e.g., area closures, catch and harvest limits, reduction of disturbance due to fishing) in the Alaska groundfish fisheries in 2003 (Bering Sea and Gulf of Alaska) around major haul-outs and rookeries within the designated critical habitat [123]. These regulations, designed to reduce competition for prey between commercial fishing and Steller sea lions and increase prey availability, are thought to have contributed to increased prey abundance and a rebound of the DPS [119,124]. In fact, counts have increased at an average of 2.17% (juveniles and adults) and 1.76% (pups) per year from 2000 to 2015, although geographical variation exists due to migration among subpopulations [118,125].

#### North Atlantic DPS of green sea turtle

The North Atlantic DPS of the green sea turtle within U.S. jurisdiction mostly nests across Florida beaches and is an ESA conservation success. The species has been increasing exponentially and has become one of the largest nesting aggregations in the western Atlantic in recent years [42]. Historically exploited across the Caribbean [54], this species has shown a high recovery potential when nesting areas are strictly protected from human disturbance and development, and fishery bycatch is substantially reduced [42]. The nesting population of green turtles across Florida showed high records of nest numbers in 2013 (36,169 nests) and 2015 (37,341 nests) compared with only 62 nests estimated in 1979 (Fig 4). These record numbers have occurred despite large annual fluctuations in nesting numbers that have been linked to changes in food supply (seagrass and macroalgae production) due to environmental changes [126]. In 2016, NMFS and USFWS reclassified green sea turtles into 11 DPSs of which the Florida nesting population was downlisted from endangered to threatened due to strong population growth and record numbers of nests in nesting beaches throughout the peninsula [127]. However, these population numbers are based on assessing the female component of the population and do not account for males and recruitment of juveniles.

ESA protections and several local and international conservation efforts have been important for the recovery of green sea turtles in the North Atlantic and other regions. ESA regulations have led to fishing gear modifications, major changes in fishing practices, time and area closures, and the establishment of turtle excluder devices for shrimp trawlers [87,128]. In particular, fishery regulations instituted because of ESA protection have been largely successful in reducing green sea turtle bycatch from Atlantic pelagic longlines and gillnets, the Chesapeake Bay pound net fishery, and the Gulf of Mexico’s shrimp and flounder trawl fisheries [42]. The ESA and other state-level laws prohibited direct harvesting of adult turtles and turtle eggs, preventing removal of mature and reproductive adults [42]. In addition, several national wildlife refuges were dedicated to protecting nesting areas on the Atlantic coast and Gulf of Mexico, with nest watchers and patrols during nesting seasons [42]. The Florida Statewide Nesting Beach Survey program, initiated in 1979 (one year after listing) as a cooperative agreement between USFWS and the Florida Fish and Wildlife Conservation Commission, now monitors ~215 nesting beaches (~825 miles) across Florida, involving federal, state, and regional institutions as well as several conservation organizations, university scientists, and private citizens [129]. Federal agencies (NMFS and USFWS) along with state agencies and other institutions have worked together in implementing the management actions in the 1991 recovery plan, eliminating or reducing threats in nesting and foraging areas [42].

## Conclusions

Recovery is occurring for representative populations of most marine mammal and sea turtle species listed under the ESA and analyzed in our study. Representative populations from species listed for over 20 years were more likely to have populations that significantly increased in numbers. In contrast, relatively recently listed species were more likely to have populations with non-significant change or declines. These findings provide critical information to set real-world expectations for recovery of marine mammal and sea turtle populations. Targeted conservation efforts triggered by ESA listing and other national and international conservation efforts have been largely successful in promoting population recovery leading to the delisting of some species and to significant increases in most others. The recovery of listed species depends ultimately on the adequate implementation of the ESA’s tools and other conservation measures. Studies have demonstrated that the government’s failure to fully implement the ESA’s protections and adequately fund conservation actions have been major impediments to species recovery [19]. In general, listed species with designated critical habitat, sufficient conservation funding, and well-implemented species-specific recovery plans tend to recover relatively faster [16,23,24]. Our analysis not only underscores the capacity of marine mammal and sea turtle populations to rebound after decades of exploitation and habitat degradation, but also highlights the success of marine species conservation through a combination of ESA protection and other conservation efforts.

## Supporting information

S1 Table Status of marine mammal and sea turtle species protected under the ESA excluded in the analyses. (PDF)

S2 Table Final model parameters used to determine location-specific population trends. (PDF)

S1 Fig Approximate geographic distribution of cetacean marine mammal populations analyzed in our study. (PDF)

S2 Fig Approximate geographic distribution of non-cetacean marine mammal populations analyzed in our study. (PDF)

S3 Fig Approximate geographic distribution of nesting beaches of sea turtle populations analyzed in our study. (PDF)

S1 Dataset Complete dataset used in the analyses. (CSV)

S1 R Code Statistical code used in exploratory analyses, modeling, and graphing. (R)

## References

[1] McCauley DJ, Pinsky ML, Palumbi SR, Estes JA, Joyce FH, Warner RR. Marine defaunation: Animal loss in the global ocean. Science (80-). 2015;347: 1255641 10.1126/science.1255641 25593191

[2] Halpern BS, Frazier M, Potapenko J, Casey KS, Koenig K, Longo C, et al Spatial and temporal changes in cumulative human impacts on the world’s ocean. Nat Commun. 2015;6: 7615 10.1038/ncomms8615 26172980 PMC4510691

[3] Pauly D, Watson R, Alder J. Global trends in world fisheries: impacts on marine ecosystems and food security. Philos Trans R Soc B Biol Sci. 2005;360: 5–12. 10.1098/rstb.2004.1574 PMC1636108 15713585

[4] Lotze HK, Lenihan HS, Bourque BJ, Bradbury RH, Cooke RG, Kay MC, et al Depletion degradation, and recovery potential of estuaries and coastal seas. Science (80-). 2006;312: 1806–1809. 10.1126/science.1128035 16794081

[5] Islam MS, Tanaka M. Impacts of pollution on coastal and marine ecosystems including coastal and marine fisheries and approach for management: a review and synthesis. Mar Pollut Bull. 2004;48: 624–649. 10.1016/j.marpolbul.2003.12.004 15041420

[6] Hoegh-Guldberg O, Bruno JF. The impact of climate change on the world’s marine ecosystems. Science (80-). 2010;328: 1523–1528. 10.1126/science.1189930 20558709

[7] Poloczanska ES, Brown CJ, Sydeman WJ, Kiessling W, Schoeman DS, Moore PJ, et al Global imprint of climate change on marine life. Nat Clim Chang. 2013;3: 919–925.

[8] Short FT, Polidoro B, Livingstone SR, Carpenter KE, Bandeira S, Bujang JS, et al Extinction risk assessment of the world’s seagrass species. Biol Conserv. 2011;144: 1961–1971.

[9] Polidoro BA, Carpenter KE, Collins L, Duke NC, Ellison AM, Ellison JC, et al The loss of species: mangrove extinction risk and geographic areas of global concern. PLoS One. 2010;5: e10095 10.1371/journal.pone.0010095 20386710 PMC2851656

[10] Carpenter KE, Abrar M, Aeby G, Aronson RB, Banks S, Bruckner A, et al One-third of reef-building corals face elevated extinction risk from climate change and local impacts. Science (80-). 2008;321: 560–563. 10.1126/science.1159196 18653892

[11] Dulvy NK, Fowler SL, Musick JA, Cavanagh RD, Kyne PM, Harrison LR, et al Extinction risk and conservation of the world’s sharks and rays. Elife. 2014;3: e00590 10.7554/eLife.00590 24448405 PMC3897121

[12] Collette BB, Carpenter KE, Polidoro BA, Juan-Jordá MJ, Boustany A, Die DJ, et al High value and long life—double jeopardy for tunas and billfishes. Science (80-). 2011;333: 291–292. 21737699 10.1126/science.1208730

[13] Webb TJ, Mindel BL. Global patterns of extinction risk in marine and non-marine systems. Curr Biol. 2015;25: 506–511. 10.1016/j.cub.2014.12.023 25639240

[14] Rohlf DJ. The Endangered Species Act: A guide to its protections and implementation. Stanford, California: Stanford Environmental Law Society; 1989.

[15] Wolf S, Hartl B, Carroll C, Neel MC, Greenwald DN. Beyond PVA: Why recovery under the Endangered Species Act is more than population viability. BioScience. 2015 pp. 200–207. 10.1093/biosci/biu218

[16] Luther D, Skelton J, Fernandez C, Walters J. Conservation action implementation, funding, and population trends of birds listed on the Endangered Species Act. Biol Conserv. 2016;197: 229–234.

[17] McCarthy DP, Donald PF, Scharlemann JPW, Buchanan GM, Balmford A, Green JMH, et al Financial costs of meeting global biodiversity conservation targets: Current spending and unmet needs. Science (80-). 2012;338: 946–949. 10.1126/science.1229803 23065904

[18] Malcom JW, Li Y-W. Data contradict common perceptions about a controversial provision of the US Endangered Species Act. Proc Natl Acad Sci. 2015;112: 15844–15849. 10.1073/pnas.1516938112 26668392 PMC4702972

[19] Schwartz MW. The performance of the Endangered Species Act. Annu Rev Ecol Evol Syst. 2008;39: 279–299. 10.1146/annurev.ecolsys.39.110707.173538

[20] U.S. Fish & Wildlife Service. Summary of listed species and recovery plans—US Fish & Wildlife Service species reports. In: U.S. Fish and Wildlife Service, ECOS Environmental Conservation Online System [Internet]. 2017. Available: http://ecos.fws.gov/tess_public/pub/Boxscore.do

[21] Scott JM, Goble DD, Davis FW. The Endangered Species Act at thirty: Vol. 2: Conserving biodiversity in human-dominated landscapes. Washington, DC: Island press; 2006. doi:1597260088, 9781597260084

[22] Male TD, Bean MJ. Measuring progress in US endangered species conservation. Ecol Lett. 2005;8: 986–992. 10.1111/j.1461-0248.2005.00806.x 34517686

[23] Taylor MFJ, Suckling KF, Rachlinski JJ. The effectiveness of the Endangered Species Act: A quantitative analysis. Bioscience. 2005;55: 360–367.

[24] Gibbs KE, Currie DJ. Protecting endangered species: do the main legislative tools work? PLoS One. 2012;7: e35730 10.1371/journal.pone.0035730 22567111 PMC3342297

[25] Suckling K, Mehrhoff LA, Beam R, Hartl B. A Wild Success: a systematic review of bird recovery under the Endangered Species Act. [Internet]. Tucson, Arizona: Center for Biological Diversity; 2016 Available: https://www.esasuccess.org/2016/report.html

[26] Miller JK, Scott JM, Miller CR, Waits LP. The endangered species act: Dollars and sense? Bioscience. 2002;52: 163–168.

[27] Ferraro PJ, McIntosh C, Ospina M. The effectiveness of the US endangered species act: An econometric analysis using matching methods. J Environ Econ Manage. 2007;54: 245–261.

[28] Dee Boersma P, Kareiva P, Fagan WF, Clark JA, Hoekstra JM. How good are endangered species recovery plans? Bioscience. 2001;51: 643–649. 10.1641/0006-3568(2001)051[0643:hgaesr]2.0.co;2

[29] Gerber LR, Hatch LT. Are we recovering? An evaluation of recovery criteria under the US Endangered Species Act. Ecol Appl. 2002;12: 668–673.

[30] Kerkvliet J, Langpap C. Learning from endangered and threatened species recovery programs: A case study using U.S. Endangered Species Act recovery scores. Ecol Econ. Elsevier; 2007;63: 499–510. 10.1016/J.ECOLECON.2006.12.007

[31] NOAA Fisheries. Endangered and threatened marine species under NMFS’ jurisdiction [Internet]. 2015. Available: http://www.nmfs.noaa.gov/pr/species/esa/listed.htm

[32] Scott JM, Goble DD, Wiens JA, Wilcove DS, Bean M, Male T. Recovery of imperiled species under the Endangered Species Act: the need for a new approach. Front Ecol Environ. 2005;3: 383–389. 10.1890/1540-9295(2005)003[0383:ROISUT]2.0.CO;2

[33] Neel MC, Leidner AK, Haines A, Goble DD, Scott JM. By the numbers: How is recovery defined by the US Endangered Species Act? Bioscience. 2012;62: 646–657. 10.1525/bio.2012.62.7.7

[34] Evans DM, Che-Castaldo JP, Crouse D, Davis FW, Epanchin-Niell R, Flather CH, et al Species recovery in the United States: Increasing the effectiveness of the Endangered Species Act. Issues Ecol. 2016;2016: 1–28.

[35] Good TP, Beechie TJ, McElhany P, McClure MM, Ruckelshaus MH. Recovery planning for Endangered Species Act-listed Pacific salmon: using science to inform goals and strategies. Fisheries. 2007;32: 426–440.

[36] Williams TH, Spence BC, Boughton DA, Johnson RC, Crozier L, Mantua N, et al Viability assessment for Pacific salmon and steelhead listed under the Endangered Species Act: Southwest. U.S. Department of Commerce, National Oceanic and Atmospheric Administration National Marine Fisheries Service Southwest Fisheries Science Center; 2016. Report No.: NOAA-TM-NMFS-SWFSC-564.

[37] Walls SC, Ball LC, Barichivich WJ, Dodd CK, Enge KM, Gorman TA, et al Overcoming challenges to the recovery of declining amphibian populations in the United States. Bioscience. 2017;67: 156–165. 10.1093/biosci/biw153

[38] Elphick CS, Reed JM, Bonta JM. Correlates of population recovery goals in endangered birds. Conserv Biol. 2001;15: 1285–1291.

[39] Leonard Jr. DL. Recovery expenditures for birds listed under the US Endangered Species Act: The disparity between mainland and Hawaiian taxa. Biol Conserv. 2008;141: 2054–2061. 10.1016/j.biocon.2008.06.001

[40] Roman J, Altman I, Dunphy-Daly MM, Campbell C, Jasny M, Read AJ. The Marine Mammal Protection Act at 40: Status, recovery, and future of U.S. marine mammals. Ann N Y Acad Sci. 2013;1286: 29–49. 10.1111/nyas.12040 23521536

[41] Mazaris AD, Schofield G, Gkazinou C, Almpanidou V, Hays GC. Global sea turtle conservation successes. Sci Adv. 2017;3: e1600730 10.1126/sciadv.1600730 28948215 PMC5606703

[42] Seminoff JA, Allen CD, Balazs GH, Dutton PH, Eguchi T, Haas H, et al. Status review of the green turtle (Chelonia mydas) under the Endangered Species Act. US Department of Commerce NOAA; 2015. Report No.: NOAA-TM-NMFS-SWFSC-539.

[43] Conant TA, Dutton PH, Eguchi T, Epperly SP, Fahy CC, Godfrey MH, et al Loggerhead sea turtle (Caretta caretta) 2009 status review under the US Endangered Species Act. Rep loggerhead Biol Rev Team to Natl Mar Fish Serv. 2009;222: 5–2.

[44] Bettridge SOM, Baker SC, Barlow J, Clapham P, Ford MJ, Gouveia D, et al Status review of the humpback whale (Megaptera novaeangliae) under the Endangered Species Act. US Department of Commerce, National Oceanic and Atmospheric Administration, National Marine Fisheries Service,[Southwest Fisheries Science Center]; 2015. Report No.: NOAA-TM-NMFS-SWFSC-540.

[45] Waples RS, Nammack M, Cochrane JF, Hutchings JA. A tale of two Acts: Endangered species listing practices in Canada and the United States. Bioscience. Oxford University Press; 2013;63: 723–734. 10.1525/bio.2013.63.9.8

[46] USFWS NMFS. Policy regarding the recognition of distinct vertebrate population segments under the Endangered Species Act. Fed Regist. 1996;61: 4722–4725. Available: https://www.gpo.gov/fdsys/pkg/FR-1996-02-07/pdf/96-2639.pdf

[47] NMFS. Endangered and Threatened Species; Identification of 14 Distinct Population Segments of the humpback whale (Megaptera novaeangliae) and revision of species-wide listing. Fed Regist. 2016;81: 62259–62320. Available: https://www.gpo.gov/fdsys/pkg/FR-2016-09-08/pdf/2016-21276.pdf

[48] Foley CM, Lynch MA, Thorne LH, Lynch HJ. Listing foreign species under the Endangered Species Act: A primer for conservation biologists. BioScience. 2017 pp. 627–637. 10.1093/biosci/bix027

[49] NMFS. Endangered and Threatened Species; Listing and recovery priority guidelines. Fed Regist. 2017;82: 24944–24950. Available: https://www.gpo.gov/fdsys/pkg/FR-2017-05-31/pdf/2017-11157.pdf

[50] Magera AM, Mills Flemming JE, Kaschner K, Christensen LB, Lotze HK. Recovery trends in marine mammal populations. PLoS One. 2013;8: e77908 10.1371/journal.pone.0077908 24205025 PMC3813518

[51] National Research Council. Assessment of sea-turtle status and trends: Integrating demography and abundance [Internet]. Washington DC: The National Acadmic Press; 2010 10.17226/12889

[52] Wallace BP, DiMatteo AD, Hurley BJ, Finkbeiner EM, Bolten AB, Chaloupka MY, et al Regional Management Units for marine turtles: A novel framework for prioritizing conservation and research across multiple scales. PLoS One. 2010;5: 1–11. 10.1371/journal.pone.0015465 21253007 PMC3003737

[53] Balazs GH, Chaloupka M. Thirty-year recovery trend in the once depleted Hawaiian green sea turtle stock. Biol Conserv. 2004;117: 491–498.

[54] McClenachan L, Jackson JBC, Newman MJH. Conservation implications of historic turtle beach loss nesting. Front Ecol Environ. 2006;4: 290–296. 10.1890/1540-9295(2006)4[290:CIOHST]2.0.CO;2

[55] Anderson DR. Model based inference in the life sciences: a primer on evidence. 1st ed. New York; London: Springer-Verlag New York; 2008.

[56] Zuur AF, Ieno EN, Walker NJ, Saveliev AA, Smith GM. Mixed effects models and extensions in ecology with R [Internet]. New York, NY; 2009 10.1007/978-0-387-87458-6

[57] Burnham KP, Anderson DR. Model selection and multi-model inference: a practical information-theoretic approach. 2nd ed Springer New York; 2002.

[58] R Core Team. R: A Language and Environment for Statistical Computing [Internet]. Vienna, Austria; 2018. Available: https://www.r-project.org/

[59] Pinheiro JC, Bates D, DebRoy S, Sarkar D. nlme: Linear and Nonlinear Mixed Effects Models. R package version 3.1–129 [Internet]. 2017. https://CRAN.R-project.org/package=nlme

[60] Hastie T, Tibshirani R. Generalized Additive Models. Stat Sci. 1986;1: 297–310. 10.1214/ss/1177013604 8548102

[61] Barton K. MuMIn: Multi-model inference. R package version 1.15.6. http://CRAN.R-project.org/package=MuMIn. 2016;

[62] Wickham H. Elegant graphics for data analysis. Media. Springer-Verlag New York; 2009;35: 211 10.1007/978-0-387-98141-3

[63] Carroll C, Vucetich JA, Nelson MP, Rohlf DJ, Phillips MK. Geography and Recovery under the U.S. Endangered Species Act. Conserv Biol. 2010;24: 395–403. 10.1111/j.1523-1739.2009.01435.x 20151988

[64] Surma S, Pitcher TJ. Predicting the effects of whale population recovery on Northeast Pacific food webs and fisheries: An ecosystem modelling approach. Fish Oceanogr. 2015;24: 291–305. 10.1111/fog.12109

[65] Roman J, Estes JA, Morissette L, Smith C, Costa D, McCarthy J, et al Whales as marine ecosystem engineers. Front Ecol Environ. 2014;12: 377–385. 10.1890/130220

[66] Perry SL, Demaster DP, Silber GK. The great whales: history and status of six species listed as endangered under the US Endangered Species Act of 1973. Mar Fish Rev Spec Issue. 1999;61: 1–74. 10.1046/j.1365-2907.2003.00005.x

[67] Baker CS, Clapham PJ. Modelling the past and future of whales and whaling. Trends Ecol Evol. 2004;19: 365–371. 10.1016/j.tree.2004.05.005 16701287

[68] Morais IOB, Danilewicz D, Zerbini AN, Edmundson W, Hart IB, Bortolotto GA. From the southern right whale hunting decline to the humpback whaling expansion: A review of whale catch records in the tropical western South Atlantic Ocean. Mamm Rev. 2017;47: 11–23.

[69] Estes JA, Burdin A, Doak DF. Sea otters, kelp forests, and the extinction of Steller’s sea cow. Proc Natl Acad Sci. 2016;113: 880–885. 10.1073/pnas.1502552112 26504217 PMC4743786

[70] Rice DW. Caribbean monk seal (Monachus tropicalis). Seals: Proceedings of a working meeting of seal specialists on threatened and depleted seals of the world, held under the auspices of the Survival Service Commission of the IUCN, IUCN Supplementary Paper. 1973. pp. 98–112.

[71] McClenachan L, Cooper AB. Extinction rate, historical population structure and ecological role of the Caribbean monk seal. Proc R Soc London B Biol Sci. 2008;275: 1351–1358. 10.1098/rspb.2007.1757 PMC2602701 18348965

[72] Lotze HK, Worm B. Historical baselines for large marine animals. Trends Ecol Evol. 2009;24: 254–262. 10.1016/j.tree.2008.12.004 19251340

[73] Johnson A, Salvador G, Kenney J, Robbins J, Kraus S, Landry S, et al Fishing gear involved in entanglements of right and humpback whales. Mar Mammal Sci. 2005;21: 635–645.

[74] Moore E, Lyday S, Roletto J, Litle K, Parrish JK, Nevins H, et al Entanglements of marine mammals and seabirds in central California and the north-west coast of the United States 2001–2005. Mar Pollut Bull. 2009;58: 1045–1051. 10.1016/j.marpolbul.2009.02.006 19344921

[75] van der Hoop JM, Vanderlaan ASM, Cole TVN, Henry AG, Hall L, Mase-Guthrie B, et al Vessel strikes to large whales before and after the 2008 ship strike rule. Conserv Lett. 2015;8: 24–32. 10.1111/conl.12105

[76] Pace RM, Corkeron PJ, Kraus SD. State-space mark-recapture estimates reveal a recent decline in abundance of North Atlantic right whales. Ecol Evol. 2017; 10.1002/ece3.3406 29152173 PMC5677501

[77] Laist DW, Shaw C. Preliminary evidence that boat speed restrictions reduce deaths of Florida manatees. Mar Mammal Sci. 2006;22: 472.

[78] Wiley DN, Moller JC, Pace RM, Carlson C. Effectiveness of voluntary conservation agreements: case study of endangered whales and commercial whale watching. Conserv Biol. 2008;22: 450–457. 10.1111/j.1523-1739.2008.00897.x 18402585

[79] Conn PB, Silber GK. Vessel speed restrictions reduce risk of collision-related mortality for North Atlantic right whales. Ecosphere. 2013;4: 1–16. 10.1890/ES13-00004.1

[80] Lewison RL, Crowder LB, Read AJ, Freeman SA. Understanding impacts of fisheries bycatch on marine megafauna. Trends Ecol Evol. 2004;19: 598–604. 10.1016/j.tree.2004.09.004

[81] Zirbel K, Balint P, Parsons ECM. Navy sonar, cetaceans and the US Supreme Court: A review of cetacean mitigation and litigation in the US. Mar Pollut Bull. 2011;63: 40–48. 10.1016/j.marpolbul.2011.03.018 21507427

[82] Waring GT, Josephson E, Maze-Foley K, Rosel PE, Editors. US Atlantic and Gulf of Mexico Marine Mammal Stock Assessments—2015. NOAA Technical Memorandum NMFS-NE-238. Woods Hole, MA; 2016.

[83] Pettis HM, Pace RM, Schick RS, Hamilton PK. North Atlantic Right Whale Consortium 2017 Annual Report Card [Internet]. 2017. Available: https://www.narwc.org/uploads/1/1/6/6/116623219/2017_report_cardfinal.pdf

[84] Kraus SD, Kenney RD, Mayo CA, McLellan WA, Moore MJ, Nowacek DP. Recent scientific publications cast doubt on North Atlantic right whale future. Front Mar Sci. Frontiers; 2016;3: 137 10.3389/fmars.2016.00137

[85] Crowder LB, Hopkins-Murphy SR, Royle JA. Effects of turtle excluder devices (TEDs) on loggerhead sea turtke strandings with imlications for conservation. Copeia. 1995;4: 773–779. 10.1017/CBO9781107415324.004

[86] Lewison RL, Crowder LB, Shaver DJ. The impact of turtle excluder devices and fisheries closures on loggerhead and Kemp’s ridley strandings in the Western Gulf of Mexico. Conserv Biol. 2003;17: 1089–1097. 10.1046/j.1523-1739.2003.02057.x

[87] Jenkins LD. Reducing sea turtle bycatch in trawl nets: a history of NMFS turtle excluder device (TED) research. Mar Fish Rev. 2012;74: 26–44.

[88] Swimmer Y, Gutierrez A, Bigelow K, Barceló C, Schroeder B, Keene K, et al Sea turtle bycatch mitigation in U.S. longline fisheries. Front Mar Sci. 2017;4 10.3389/fmars.2017.00260

[89] Nester LR. Effects of off-road vehicles on the nesting activity of loggerhead sea turtles in North Carolina. PhD Thesis, University of Florida. 2006.

[90] Salmon M. Protecting sea turtles from artificial night lighting at Florida’s oceanic beaches Ecological consequences of artificial night lighting. C. Rich an. Washington DC: Island Press; 2006 pp. 141–168.

[91] NMFS USFWS. Leatherback sea turtle (Dermochelys coriacea) 5-year review: Summary and evaluation. Silver Spring, Maryland and Jacksonville, Florida: National Marine Fisheries Service, Office of Protected Resources and U.S. Fish and Wildlife Service Southeast Region; 2013.

[92] Stewart KR, Martin KJ, Johnson C, Desjardin N, Eckert SA, Crowder LB. Increased nesting, good survival and variable site fidelity for leatherback turtles in Florida, USA. Biol Conserv. Elsevier Ltd; 2014;176: 117–125. 10.1016/j.biocon.2014.05.008

[93] Caillouet CW Jr., Shaver DJ, Landry AM Jr.. Kemp’s Ridley sea turtle (Lepidochely kempii) head-start and reintroduction to Padre Island National Seashore, Texas. Herpetol Conserv Biol. 2015;10: 309–377.

[94] Humber F, Godley BJ, Broderick AC. So excellent a fishe: A global overview of legal marine turtle fisheries. Divers Distrib. 2014;20: 579–590. 10.1111/ddi.12183

[95] Hamann M, Godfrey MH, Seminoff JA, Arthur K, Barata PCR, Bjorndal KA, et al Global research priorities for sea turtles: Informing management and conservation in the 21st century. Endanger Species Res. 2010;11: 245–269. 10.3354/esr00279

[96] U.S. Fish and Wildlife Service. Southwest Alaska Distinct Population Segment of the Northern Sea Otter (Enhydra lutris kenyoni)- Recovery Plan [Internet]. Anchorage, Alaska; 2013. Available: http://www.adfg.alaska.gov/static/species/specialstatus/pdfs/seaotter_2010_draft_recovery_plan.pdf

[97] NMFS (National Marine Fisheries Service). Recovery plan for the Cook Inlet beluga whale. Alaska Region, Protected Resources Division, Juneau, AK: National Marine Fisheries Service; 2017.

[98] Waring GT, Josephson E, Maze-Foley K, Rosel PE. US Atlantic and Gulf of Mexico marine mammal stock assessments–2014. Fin Whale (Balaenoptera physalus): Western North Atlantic Stock. NOAA Tech Memo NMFS NE. 2015;231: 39–45.

[99] van Houtan KS, Halley JM. Long-term climate forcing in loggerhead sea turtle nesting. PLoS One. 2011;6: e19043 10.1371/journal.pone.0019043 21589639 PMC3083431

[100] NOAA Fisheries. Species in the spotlight: Survive to thrive [Internet]. 2016. Available: https://www.fisheries.noaa.gov/topic/endangered-species-conservation#species-in-the-spotlight

[101] Holt MM, Veirs V, Veirs S. Noise effects on the call amplitude of Southern Resident killer whales (Orcinus orca). Bioacoustics. 2008;17: 164–166. 10.1080/09524622.2008.9753802

[102] Lusseau D, Bain DE, Williams R, Smith JC. Vessel traffic disrupts the foraging behavior of southern resident killer whales Orcinus orca. Endanger Species Res. 2009;6: 211–221. https://doi-org/10.3354/esr00154

[103] Hanson B, Ward E, Ford M, O’Neill S, Balcomb K. Factors affecting Southern Resident killer whale growth and recovery. Salish Sea Ecosyst Conf. 2014;

[104] O’Neill S, Ylitalo G, West J. Energy content of Pacific salmon as prey of northern and southern resident killer whales. Endanger Species Res. 2014;25: 265–281. 10.3354/esr00631

[105] Matkin CO, Moore MJ, Gulland FM. Review of recent research on Southern Resident killer whales to detect evidence of poor body condition in the population [Internet]. Woods Hole, MA: Independent Science Panel; 2017 10.1575/1912/8803

[106] Baker JD, Polovina JJ, Howell EA. Effect of variable oceanic productivity on the survival of an upper trophic predator, the Hawaiian monk seal Monachus schauinslandi. Mar Ecol Prog Ser. 2007;346: 277–283.

[107] Ford JKB, Ellis GM, Olesiuk PF, Balcomb KC. Linking killer whale survival and prey abundance: food limitation in the oceans’ apex predator? Biol Lett. 2010;6: 139–142. 10.1098/rsbl.2009.0468 19755531 PMC2817236

[108] Norman SA, Hobbs RC, Goertz CE, Burek-Huntington KA, Shelden KE, Smith WA, et al Potential natural and anthropogenic impediments to the conservation and recovery of Cook Inlet beluga whales, Delphinapterus leucas. Mar Fish Rev. 2015;77: 89–105.

[109] Wasser SK, Lundin JI, Ayres K, Seely E, Giles D, Balcomb K, et al Population growth is limited by nutritional impacts on pregnancy success in endangered Southern Resident killer whales (Orcinus orca). PLoS One. 2017;12: e0179824 10.1371/journal.pone.0179824 28662095 PMC5491047

[110] National Marine Fisheries Service (DFO). Recovery plan for Southern Resident killer whales (Orcinus orca). Seatle, WA: National Marine Fisheries Service; 2008; 251.

[111] National Marine Fisheries Service. Main Hawaiian Islands monk seal management plan. [Internet]. Honolulu, HI: National Marine Fisheries Service, Protected Resources Division Pacific Islands Regional Office; 2016. Available: https://www.fpir.noaa.gov/Library/PRD/Hawaiian monk seal/HMS_Management_Plan_FNL.pdf

[112] NMFS. Protective regulations for killer whales in the Northwest region under the Endangered Species Act and Marine Mammal Protection Act. Fed Regist. 2011;76: 20870–20890. Available: https://www.gpo.gov/fdsys/pkg/FR-2011-04-14/pdf/2011-9034.pdf

[113] NMFS. Recovery plan for the Hawaiian monk seal (Monachus schauinslandi) revision original version: March 1983. Honolulu, HI: National Marine Fisheries Service, Protected Resources Division Pacific Islands Regional Office; 2007.

[114] NMFS (National Marine Fisheries Service). Monk seal population size and threats: NOAA Fisheries Pacific Islands Regional Office [Internet]. 2017. Available: https://www.fpir.noaa.gov/PRD/prd_hms_population_threats.html

[115] NMFS. Regulations governing the approach to humpback whales in Alaska. Fed Regist. 2001;66: 29502–29509. Available: https://www.gpo.gov/fdsys/pkg/FR-2001-05-31/pdf/01-13677.pdf

[116] NMFS. Endangered fish or wildlife; special prohibitions; approaching humpback whales in Hawaiian waters. Fed Regist. 1995;60: 3775–3776. Available: https://www.gpo.gov/fdsys/pkg/FR-1995-01-19/pdf/95-1340.pdf

[117] NMFS. Hawaiian Islands humpback whale National Marine Sanctuary regulations. Fed Regist. 1995;60: 48000–48010. Available: https://www.gpo.gov/fdsys/pkg/FR-1995-09-15/pdf/95-22997.pdf

[118] Fritz LW, Sweeney KM, Towell RG, Gelatt TS. Aerial and ship-based surveys of Steller Sea lions (Eumetopias jubatus) conducted in Alaska in June-July 2013 through 2015, and an update on the status and trend of the Western Distinct Population Segment in Alaska. Alaska Fisheries Science Center; 2016. Report No.: NMFS-AFSC-321.

[119] Muto MM, Helker VT, Angliss RP, Allen BA, Boveng PL, Breiwick JM, et al. Alaska Marine Mammal Stock Assessments, 2016: Steller lion (Eumetopias jubatus): Western U.S. Stock [Internet]. Anchorage, Alaska; 2016. Report No.: NOAA-TM-AFSC-355. Available: https://www.fisheries.noaa.gov/webdam/download/76143333

[120] Sease JL, Taylor WP, Loughlin TR, Pitcher KW. Aerial and land-based surveys of Steller sea lions (Eumetopias jubatus) in Alaska, June and July 1999 and 2000 [Internet]. Alaska Fisheries Science Center: U.S. DEPARTMENT OF COMMERCE National Oceanic and Atmospheric Administration; 2001 Report No.: NMFS-AFSC-122. Available: https://www.afsc.noaa.gov/Publications/AFSC-TM/NOAA-TM-AFSC-122.pdf

[121] Sease JL, Gudmundson C.J. Aerial and Land-Based Surveys of Steller Sea Lions (Eumetopias jubatus) From the Western Stock in Alaska, June and July 2001 and 2002 [Internet]. NOAA Technical Memorandum. Anchorage, Alaska; 2002 Available: https://www.afsc.noaa.gov/publications/AFSC-TM/NOAA-TM-AFSC-131.pdf

[122] Fritz L, Lynn M, Kunisch E, Sweeney K. Aerial, ship and land-based surveys of Steller sea lions (Eumetopias jubatus) in Alaska, June and July 2005–2007. NOAA Tech Memo NMFS-AFSC. 2008;183: 81.

[123] NMFS. Fisheries of the Exclusive Economic Zone off Alaska; Steller sea lion protection measures for the groundfish fisheries off Alaska. Fed Regist. 2003;68: 204–236. Available: https://www.gpo.gov/fdsys/pkg/FR-2003-01-02/pdf/02-32844.pdf

[124] Hui TCY, Gryba R, Gregr EJ, Trites AW. Assessment of competition between fisheries and steller sea lions in Alaska based on estimated prey biomass, fisheries removals and predator foraging behaviour. PLoS One. 2015;10: e0123786 10.1371/journal.pone.0123786 25950178 PMC4424003

[125] Johnson DS, Fritz L. agTrend: A Bayesian approach for estimating trends of aggregated abundance. Methods Ecol Evol. 2014;5: 1110–1115.

[126] Broderick AC, Godley BJ, Hays GC. Trophic status drives interannual variability in nesting numbers of marine turtles. Proc R Soc B Biol Sci. 2001;268: 1481–1487. 10.1098/rspb.2001.1695 11454292 PMC1088767

[127] NMFS. Endangered and Threatened Wildlife and Plants; Final rule to list eleven Distinct Population Segments of the green sea turtle (Chelonia mydas) as endangered or threatened and revision of current listings under the Endangered Species Act. Fed Regist. 2016;81: 20057–20090. Available: https://www.gpo.gov/fdsys/pkg/FR-2016-04-06/pdf/2016-07587.pdf

[128] Finkbeiner EM, Wallace BP, Moore JE, Lewison RL, Crowder LB, Read AJ. Cumulative estimates of sea turtle bycatch and mortality in USA fisheries between 1990 and 2007. Biol Conserv. 2011;144: 2719–2727.

[129] FWC. Sea Turtle Monitoring (the SNBS and INBS Programs) [Internet]. 2017 [cited 17 Sep 2017]. Available: http://myfwc.com/research/wildlife/sea-turtles/nesting/monitoring/

