# Supplementary material for: Marine mammals and sea turtles listed under the U.S. Endangered Species Act are recovering
Source: PLoS One. 2019 Jan 16;14(1):e0210164. doi: 10.1371/journal.pone.0210164 (PMC6334928; doi:10.1371/journal.pone.0210164)
Supplement: S1 Table — (PDF) [file pone.0210164.s001.pdf]

S1 Table. Status of marine mammal and sea turtle species protected under the ESA excluded in the analyses. These species inhabit foreign waters (F), have been listed after 2012, or there was not reliable population abundance data to estimate trends. Distinct population segment (DPS), marine mammal stock (Stock), or entire species listed (n/a); year of first listed; ESA status as endangered (E), threatened (T), delisted (D), or status change (e.g., T→E); and number of years listed are shown. Year of ESA status change due to down-listing (dl) and up-listing (ul); and reason for delisting such as recovered (re) and extinction (ex) are presented. Several species were listed before 1973 under the Endangered Species Preservation Act of 1966, and the Endangered Species Conservation Act of 1969, which were later replaced by the more comprehensive Endangered Species Act of 1973. Data as of July 2017 (NOAA Fisheries 2017).

Common Name	Scientific Name	DPS/Stock/Population	Water	Listed year	ESA status	Years listed	Status change
**Mammal: Cetacea**							
Baiji[1]	*Lipotes vexillifer*	n/a	F	1989	E	28	–
Indus River dolphin	*Platanista gangetica minor*	n/a	F	1991	E	26	–
Vaquita[2]	*Phocoena sinus*	n/a	F	1985	E	32	–
False killer whale	*Pseudorca crassidens*	Main Hawaiian Islands Insular	US	2012	E	5	
Humpback whale	*Megaptera novaeangliae*	Arabian Sea DPS	F	1970	E	47	–
		Brazil DPS	F	1970	T→D	46	2016 –re
		Cape Verde Islands/NW Africa DPS	F	1970	E	47	–
		East Australia DPS	F	1970	T→D	46	2016 –re
		Oceania DPS	US/F	1970	E→D	46	2016 –re
		Southeast Africa DPS	F	1970	T→D	46	2016 –re
		Southeastern Pacific DPS	F	1970	T→D	46	2016 –re
		Southwest Africa DPS	F	1970	T→D	46	2016 –re
		West Australia DPS	F	1970	T→D	46	2016 –re
		Western North Pacific DPS	US/F	1970	E	47	–
Southern right whale	*Eubalaena australis*	n/a	F	1970	E	47	–
N. Pacific right whale	*Eubalaena japonica*	Eastern North Pacific Stock	US/F	1970	E	47	–
Sperm whale	*Physeter macrocephalus*	n/a	US/F	1970	E	47	–
**Mammal: Carnivora**							
Polar bear	*Ursus maritimus*	n/a	US/F	2008	T	9	–
Caribbean monk seal	*Monachus tropicalis*	n/a	US/F	1967	E→D	41	2008 –ex
Mediterranean monk seal	*Monachus monachus*	n/a	F	1970	E	47	–
Ringed seal[3]	*Phoca hispida saimensis*	Saimaa DPS	F	1993	E	24	–

	Phoca hispida botnica	Baltic DPS	F	2012	T	5	–
	Phoca hispida ladogensis	Ladoga DPS	F	2012	E	5	–
	Phoca hispida ochotensis	Okhotsk DPS	F	2012	T	5	–
Spotted seal	*Phoca largha*	Southern DPS	F	2010	T	7	–
**Reptile: Sea Turtles**							
Green turtle	*Chelonia mydas*	Central South Pacific DPS	US/F	1978	T→E	39	2016 –ul
		East India-West Pacific DPS	F	1978	T	39	–
		East Pacific DPS	F	1978	E→T	39	2016 –dl
		Mediterranean DPS	F	1978	T→E	39	2016 –ul
		North Indian DPS	F	1978	T	39	–
		Southwest Indian DPS	F	1978	T	39	–
		Southwest Pacific DPS	F	1978	T	39	–
Hawksbill turtle	*Eretmochelys imbricata*	n/a Hawaii Population	US/F	1970	E	47	–
		n/a Guam Population	US/F	1970	E	47	–
Leatherback turtle [4]	*Dermochelys coriacea*	n/a (Pacific region)	US/F	1970	E	47	–
Loggerhead turtle	*Caretta caretta*	Mediterranean Sea DPS	F	1978	T→E	39	2011–ul
		Northeast Atlantic Ocean DPS	F	1978	T→E	39	2011–ul
		North Indian Ocean DPS	F	1978	T→E	39	2011–ul
		North Pacific Ocean DPS	US/F	1978	T→E	39	2011–ul
		South Atlantic Ocean DPS	F	1978	T	39	–
		South Pacific Ocean DPS	F	1978	T→E	39	2011–ul
		Southeast Indo-Pacific Ocean DPS	F	1978	T	39	–
		Southwest Indian Ocean DPS	F	1978	T	39	–
Olive ridley turtle[5]	*Lepidochelys olivacea*	(Mexico's Pacific coast)	US/F	1978	E	39	–
		(All other areas)	US/F	1978	T	39	–

[1] Also known as Chinese River dolphin, is likely extinct. The last confirmed photographic sighting was in 2002 (Turvey et al. 2007).

[2] Also known as Gulf of California harbor porpoise will likely go extinct in 2018 unless fishery bycatch is eliminated (Taylor et al. 2016).

[3] On March 11, 2016, the U.S. District Court for the District of Alaska issued a decision vacating NOAA Fisheries' December 28, 2012, listing of the Arctic ringed seal as threatened. A notice of appeal of the District Court decision was filed on May 3, 2016, and ESA status may be returned to the Arctic ringed seal.

[4] The Pacific population of leatherback was excluded from the analysis because nesting occurs in foreign beaches. Foraging may occur in U.S. waters.

[5] The olive ridley sea turtle is managed as two population groups: Mexico's Pacific coast and all other populations. Both populations were excluded from the analysis because nesting occurs in foreign beaches. Foraging may occur in U.S. waters.

References

NOAA Fisheries. 2017. Endangered and Threatened Marine Species: NOAA Fisheries. Available from http://www.nmfs.noaa.gov/pr/species/esa/ (accessed May 24, 2017).

Taylor BL et al. 2016. Extinction is Imminent for Mexico's Endemic Porpoise Unless Fishery Bycatch is Eliminated. Conservation Letters. Available from http://onlinelibrary.wiley.com/doi/10.1111/conl.12331/abstract (accessed April 11, 2017).

Turvey ST et al. 2007. First human-caused extinction of a cetacean species? Biology letters **3**:537–540.
