# Supplementary material for: Marine mammals and sea turtles listed under the U.S. Endangered Species Act are recovering
Source: PLoS One. 2019 Jan 16;14(1):e0210164. doi: 10.1371/journal.pone.0210164 (PMC6334928; doi:10.1371/journal.pone.0210164)
Supplement: S2 Table — (PDF) [file pone.0210164.s002.pdf]

S2 Table. Final model parameters used to determining location-specific population trends. Model type consisted of linear model (*lm*), generalized linear model (*glm*), generalized least squatted model (*gls*), or generalized additive model (*gam*). Abundance was log transformed in some instances and indicated with a (log) next to model type. Family (link) is the description of the error distribution and link function used in the models. Correlation and variance structures were used only for *gls* models to account for temporal autocorrelation and heteroscedasticity structure, respectively. P-values for each model are shown. Final models were obtained through an iterative model selection using AICc.

Common Name	DPS/Stock/Pop	Pop. Trend (↑→↓)	Model type	Family (link)	Variance Structure	Correlation structure	P-value
**Cetacean**							
Beluga whale	Cook Inlet DPS	→	*glm*	Poisson (log)	–	–	0.17900
Blue whale	Eastern North Pacific Stock	↑	*gam*	Poisson (identity)	–	–	0.00506
Bowhead whale	Western Arctic Stock	↑	*gls*	Gaussian	–	corAR1	<0.00001
Fin whale	CA, OR, WA Stock	↑	*gls*	Gaussian	–	–	<0.00005
Fin whale	Western North Atlantic Stock	→	*gls*	Gaussian	–	–	0.61690
Grey whale	Eastern North Pacific Stock – *d.*	↑	*gam*	Gaussian (log)	–	–	0.00144
Grey whale	Western North Pacific Stock	↑	*gam*	Gaussian (log)	–	–	<0.00001
Humpback whale	Central America DPS (CA/OR)	↑	*gls*	Gaussian	varExp	corAR1	<0.00001
Humpback whale	Hawaii DPS – *d.*	↑	*gam*	Gamma (log)	–	–	0.00004
Humpback whale	Mexico DPS (SE AK – AK P.)	↑	*gls*	Gaussian	varPower	corAR1	<0.00001
Humpback whale	West Indies DPS – *d.*	↑	*gls*	Gaussian	varPower	corAR1	0.00160
Killer whale	Southern Resident DPS	↓	*glm*	Gaussian (identity)	–	–	0.00024
N. Atl. right whale	North Atlantic – (90-10)	↑	*gam*	Gamma (log)	–	–	<0.00001
Sei whale	Eastern North Pacific Stock	↑	*gam*	Gaussian (log)	–	–	0.00087
Sei whale	Nova Scotia Stock	↑	*gls*	Gaussian	varFixed	corAR1	0.00210
**Carnivora**							
Guadalupe fur seal	Guadalupe Island, Mexico	↑	*gls*	Gaussian (identity)	varIndt	corAR1	<0.00001
Hawaiian monk seal	NW Hawaiian Islands – (85-13)	↓	*gls*	Gaussian (identity)	varPower	corLin	<0.00001
	NW Hawaiian Islands – (13-16)	↑	*glm*	Gaussian (log)	–	–	0.01650
Northern sea otter	Southwest Alaska DPS	→	*glm*	Gaussian (log)	–	–	0.08300

Southern sea otter	California	↑	*gls*	Gaussian	varPower	corAR1	<0.00001
Steller sea lion	Eastern DPS (CA-SEAK)	↑	*gls*	Gaussian (identity)	varExp	–	<0.00001
Steller sea lion	Western DPS – (90-03)	↓	*gam*	Gaussian (log)	–	–	<0.00001
	Western DPS – (03-15)	↑	*gam*	Gaussian (log)	–	–	<0.00001
**Sirenia**							
Florida manatee	Florida	↑	*glm* (log)	Gamma (log)	–	–	<0.00001
Antillean manatee	Puerto Rico	↑	*glm* (log)	Gamma (log)	–	–	0.00012
**Sea Turtles**							
Green turtle	Central North Pacific DPS (East Island, French Frigate, HI)[1]	↑	*glm* (log)	Gaussian (identity)	–	–	<0.00001
Green turtle	Central West Pacific DPS (Guam waters)[2]	→	*gam* (log)	Gaussian (identity)	–	–	0.13630
Green turtle	North Atlantic DPS (Florida index beaches)[3]	↑	*glm* (log)	Gaussian (identity)	–	–	<0.00001
Green turtle	South Atlantic DPS (Buck Reef NWR + Sandy Point NWR + Jack, Isaac, and East End Bays)[1]	↑	*gam* (log)	Gaussian (identity)	–	–	<0.00001
Hawksbill turtle	Atlantic DPS (Mona Island, Puerto Rico)[3]	↑	*gam* (log)	Gaussian (identity)	–	–	<0.00001
Kemp's Ridley turtle	Texas[3] – *(since 1980)*	↑	*gam* (log)	Gamma (log)	–	–	<0.00001
Leatherback turtle	Atlantic DPS (Florida +Puerto Rico + Sandy Point NWR, VI)[3]	↑	*gam* (log)	Gaussian (log)	–	–	<0.00001
Loggerhead turtle	Northwest Atlantic DPS (Peninsular FL index beaches)[3]	→	*gam*	Gaussian (identity)	–	–	0.11360

[1] Number of nesting females
[2] Number of individuals
[3] Number of nests
