# Supplementary material for: Marine mammals and sea turtles listed under the U.S. Endangered Species Act are recovering
Source: PLoS One. 2019 Jan 16;14(1):e0210164. doi: 10.1371/journal.pone.0210164 (PMC6334928; doi:10.1371/journal.pone.0210164)
Supplement: S3 Fig — (PDF) [file pone.0210164.s005.pdf]

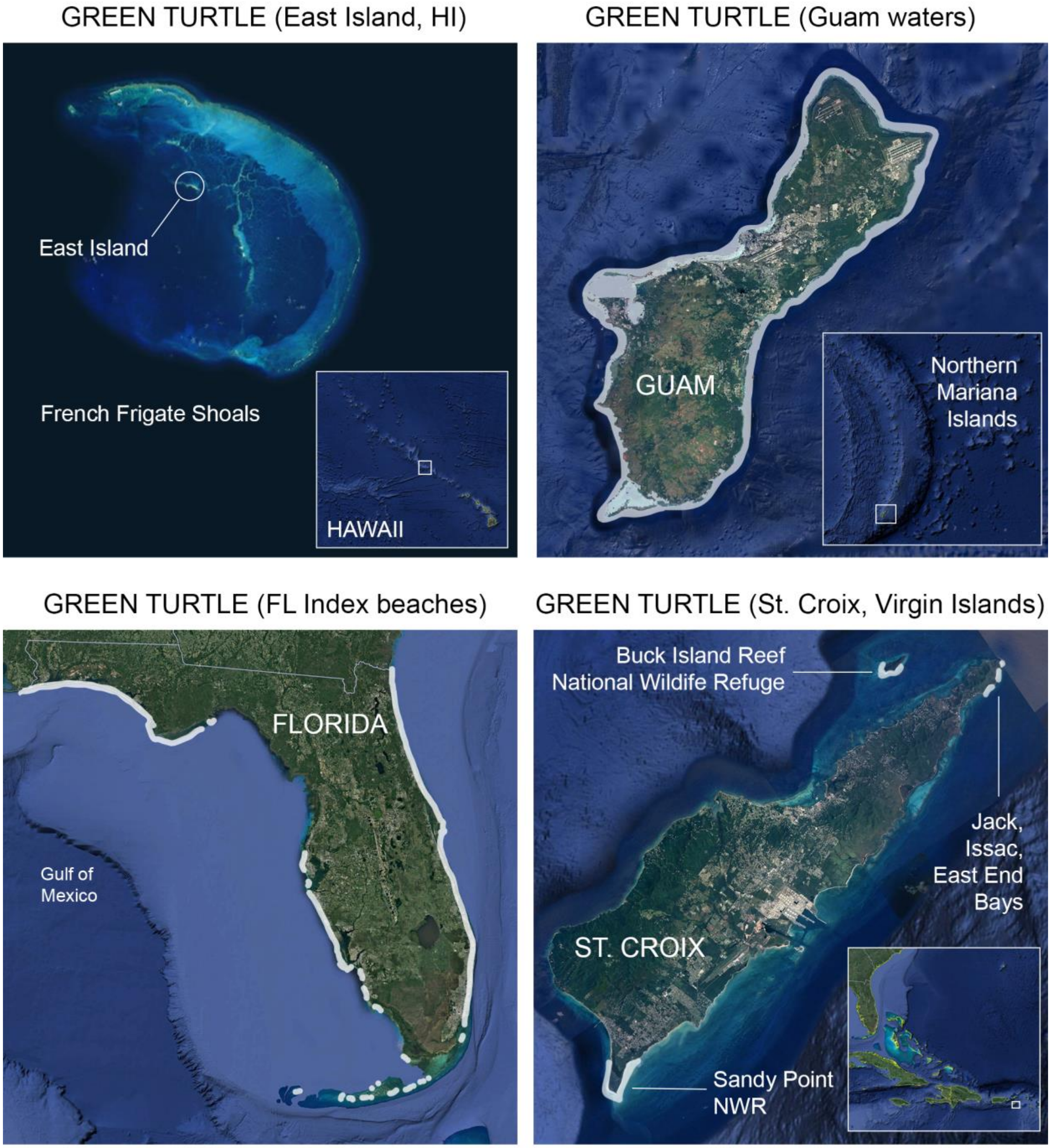

S3 Figure. Approximate geographic distribution (shaded area) of nesting beaches of sea turtle populations analyzed in our study (*continue in next page…*).

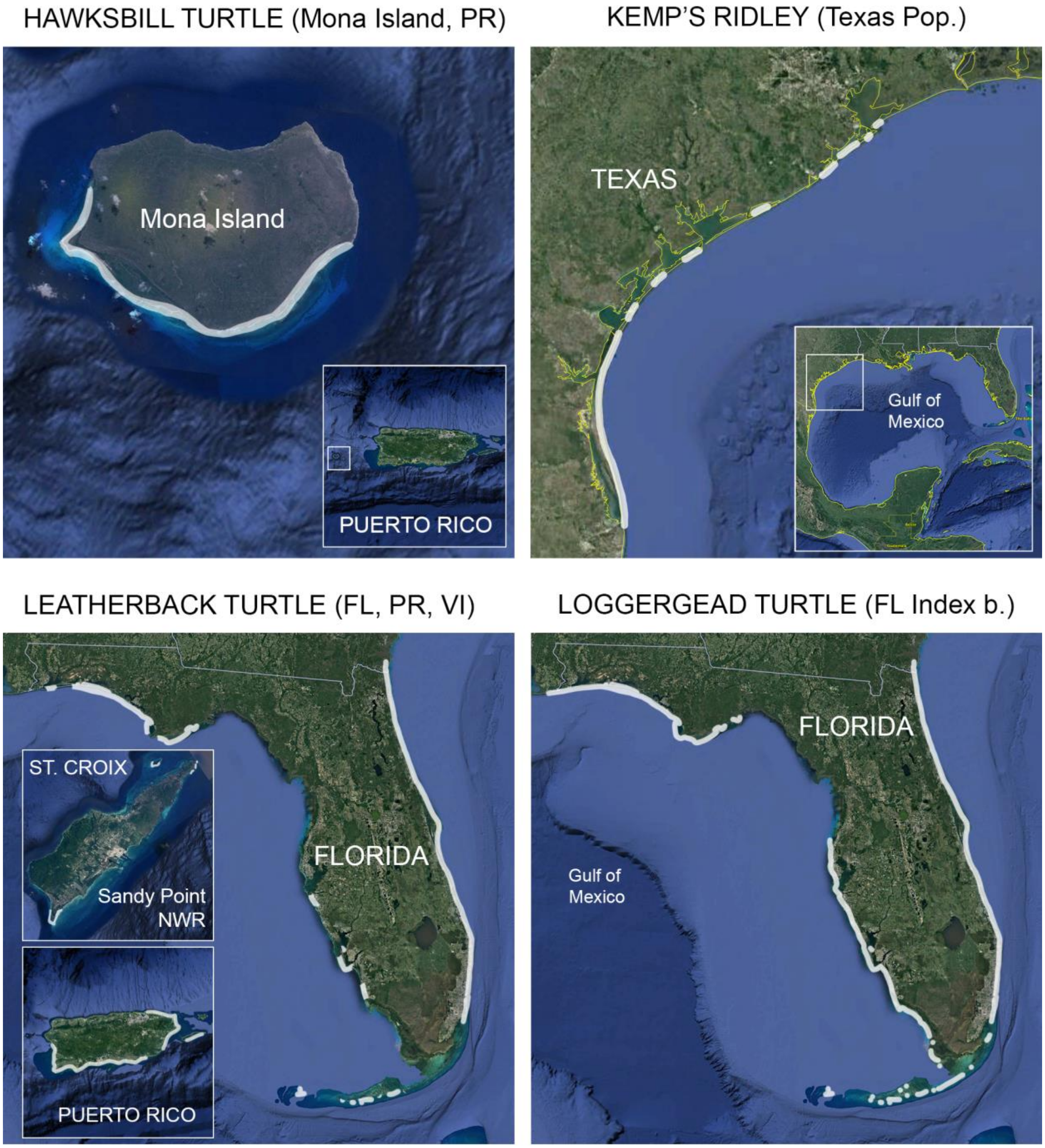

S3 Figure. *(cont.)* Approximate geographic distribution (shaded area) of nesting beaches sea turtle populations analyzed in our study.
